# Supplementary material for: Flexopiezoelectricity at ferroelastic domain walls in WO3 films
Source: Nat Commun. 2020 Sep 29;11:4898. doi: 10.1038/s41467-020-18644-w (PMC7524836; doi:10.1038/s41467-020-18644-w)
Supplement: Supplementary file 1 — Supplementary Information [file 41467_2020_18644_MOESM1_ESM.pdf]

Supplementary Information for

Flexopiezoelectricity at ferroelastic domain walls in WO_3 films

Yun *et al.*

This PDF file includes:

Supplementary Figures 1–23

Supplementary Table 1

Supplementary Notes

1. Structural analysis by TEM
2. Estimated strain gradient at the center of ferroelastic domain wall
3. PFM measurement and calibration of piezoelectric response
4. Phenomenological description of the flexopiezoelectricity
5. Domain wall width and large-area strain by plane-view TEM analysis
6. Strain gradient and domain wall width measured from X-ray diffraction
7. Flexoelectric polarizations at the center of ferroelastic domain wall
8. Phase field simulation
9. Detection of depolarized fields using DPC-STEM
10. Ferroelastic domain wall widths for different film thicknesses

Supplementary References 1–10

Supplementary Note 1. Structural analysis by TEM

To clearly understand the ferroelastic twin structure, we obtained dark-field TEM images using various g-beams (Supplementary Fig. 1) and the electron diffraction patterns of domains A and B. They were separately measured by using the smallest selected area (SA) aperture, and then compared with the simulated patterns constructed by CrystalMaker software based on the space group $P2_1/n$ (monoclinic, No. 14) with the lattice parameters and atomic positions of γ-WO_3 phase[1] (Supplementary Fig. 2).

As described in the main text, the twin domain walls in WO_3 were inclined 45° with respect to the in-plane $[1\bar{1}0]$ and [001] directions of $YAlO_3$ substrate, and the specimen for TEM analysis was prepared thicker than the typical TEM specimens to minimize the domain relaxation. Therefore, domains in the cross-sectional TEM can partially overlap with different domains along the viewing direction. In addition, since the width of the domains is not constant as shown in Supplementary Fig. 2, it is highly likely that the diffraction patterns will be overlapped and/or interfered. Although we used the smallest SA aperture about 150 nm in diameter to acquire the diffraction pattern in each domain, the projected diffraction pattern obtained in one domain may slightly overlap with the diffraction from the neighboring domains. Also, we cannot exclude contributions from the macro-domain wall regions and/or interference effects of non-uniform atomic arrangements. Because of this, some weak forbidden reflections are observed in the experimental diffraction patterns (as indicated by dashed circles). However, the strong reflections indicated by the red, orange, and yellow colored circles in the experimental diffraction patterns of Supplementary Figs. 2c and d result from the proper crystallographic symmetry, and they are consistent with the simulated ones.

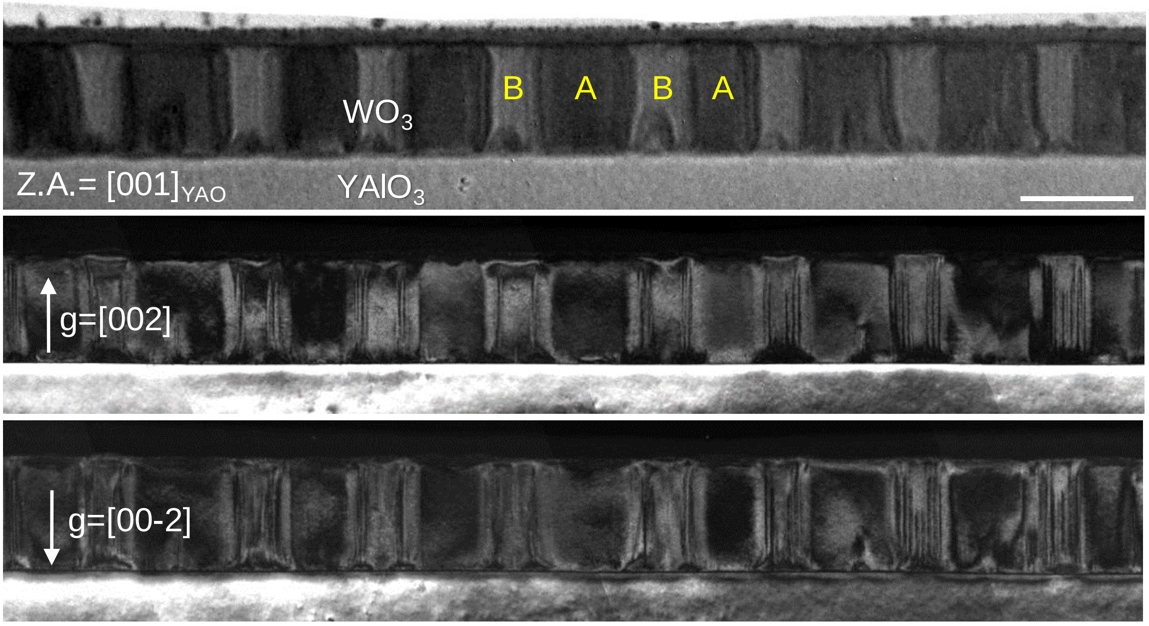

Supplementary Fig. 1 | BF-TEM image taken along zone axis $[001]_{YAO}$ and DF-TEM images under different g-beam conditions. The ferroelastic macro domains of WO_3 film are tilt by ±45° relative to $[001]_{YAO}$ zone axis. Two-beam DF-TEM images with g=[002], g=[00-2] show the domain structure more clearly. Scale bar represents 300 nm.

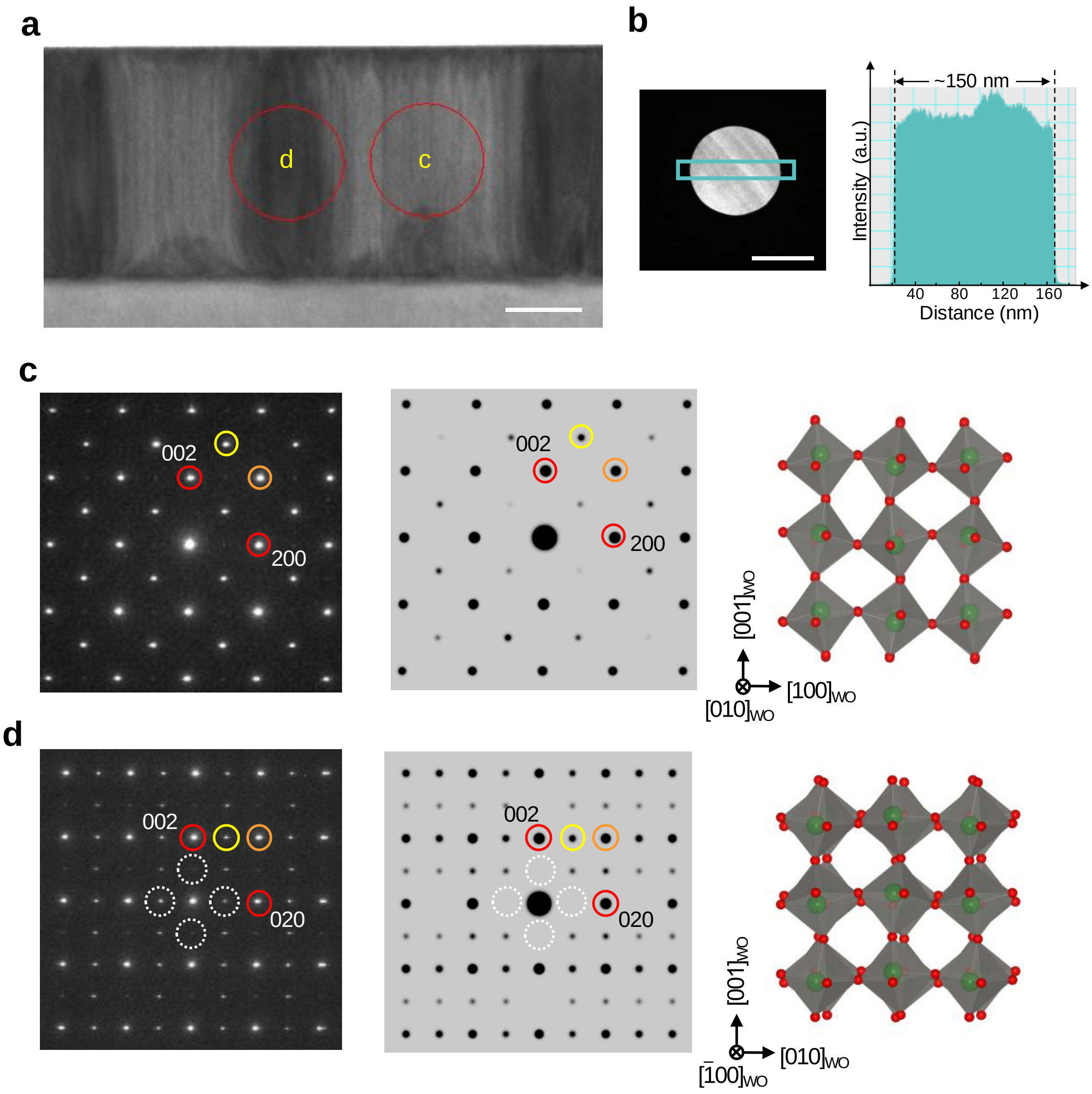

Supplementary Fig. 2 | Simulation of electron diffraction patterns of WO_3. a, The cross sectional BF-TEM image taken along the $[1\bar{1}0]_{YAO}$. **b**, TEM image showing the size of selected area (SA) aperture. The smallest SA aperture was used for domain structure analysis and its diameter is about 150 nm. **c**, Experimental (left) and simulated electron diffraction pattern (center) taken along the [010] direction of WO_3 monoclinic structure (right). **d**, Experimental (left) and calculated electron diffraction pattern (center) taken along the $[\bar{1}00]$ of WO_3 monoclinic structure (right). Scale bars indicate 100 nm.

Supplementary Note 2. Estimated strain gradient at the center of ferroelastic domain wall

The surface topographic image of a ~ 610 nm-thick WO_3 film (Fig. 1a) shows a hierarchical twin structure on the background of a step-terrace structure with single-unit-cell (less than 4 Å) steps that indicates the film was grown in a step-flow growth mode. It is remarkable that such large-thickness film still has an atomically flat surface to the extent that the peaks and troughs of fine domains due to the mosaic rotations ($\pm 0.82^{\circ}$) are clearly seen. The AFM sensitivity along the normal is quite good enough to see atomic scale height changes, so we can say that the topographic modulation is tiny in reality and negligible compared with the lateral length scale and film thickness. In Fig. 1a, somewhat irregular step edges look close to the macro-domain walls (straight along the dashed lines which are defined along the meeting points of the A and B domains) and the step edges seem to influence on the macro-domain walls nearby. However, the effect was not significant and the similar alignment happened by accident. The step edge directions and widths are determined by uncontrolled misaligned cutting of the substrate (~ 0.1°), so we can find many counter examples where the correlations between macro-domain walls and step edges are weak.

Based on reasoning, the unit-cell deformations between the A and B domains are plotted in the top view (Supplementary Fig. 3). To quantitatively consider the strain and strain gradient at the interfaces, we deal with the second-rank strain tensor. The compatibility between the A and B domains induces the local unit cells to tilt slightly in the global coordinate axis by about $\pm 0.4^{\circ}$, but the angle negligibly affects the following description. First, we build the strain tensor in accordance with the lattice parameters of monoclinic unit cell in the *xyz* coordinate compatible with the crystal axes and then perform a coordinate transformation to obtain the representation in the $x'y'z'$ coordinate system that is appropriate for the ferroelastic domain wall geometry (*i.e.*, x' is perpendicular to the ferroelastic domain wall, y' is along the wall, and z' is identical to z).

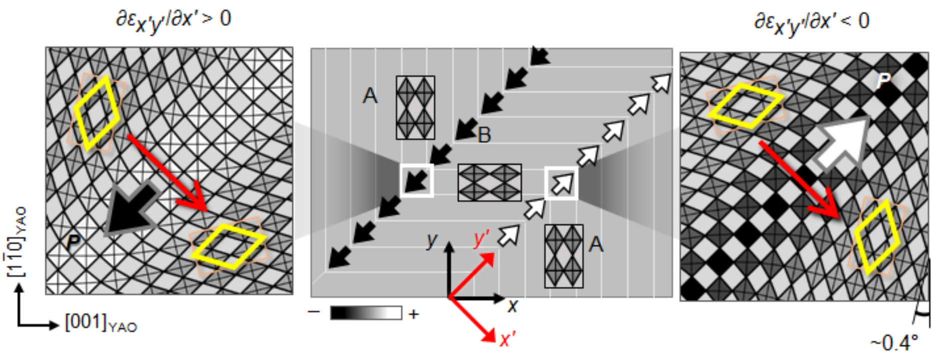

Supplementary Fig. 3 | Schematics for the expected strain gradients at ferroelastic domain walls and induced piezoresponses. The middle image shows a ferroelastic domain structure with projected monoclinic unit cells of A and B domains (grey boxes with four octahedra). Black and white arrows represent the induced flexoelectric polarizations expected from the strain gradients at ferroelastic domain walls. Grey scale bars represent the direction and magnitude of the strain gradient. In the left and right images, detailed strain gradients are described across the ferroelastic domain walls. Yellow boxes stand for the rhombic ($\sqrt{2} \times \sqrt{2}$) unit cells parallel to y'-axis.

In Supplementary Fig. 4, a top-view schematic of hierarchical domain structure is shown with the exaggerated tilt angle of local domain axis compared to global *x*-*y* axis (the angle is ±0.4°, which is negligibly small). Four-variant monoclinic unit cells (A_1, A_2, B_1, or B_2) are exhibited. Since we are interested in strain gradients at ferroelastic domain walls between A and B domains across the grey arrows, the second-rank strain tensors are needed to be transformed to *x*'-*y*' axes, which is parallel and perpendicular to the 45° ferroelastic domain walls (In the followings, the parameters and matrixes in red are represented in the *x*'*y*'*z*' coordinate.),

$$\varepsilon = \begin{pmatrix} \varepsilon_{xx} & \varepsilon_{xy} & \varepsilon_{xz} \\ \varepsilon_{yx} & \varepsilon_{yy} & \varepsilon_{yz} \\ \varepsilon_{zx} & \varepsilon_{zy} & \varepsilon_{zz} \end{pmatrix} \rightarrow \varepsilon' = \begin{pmatrix} \varepsilon_{x'x'} & \varepsilon_{x'y'} & \varepsilon_{x'z'} \\ \varepsilon_{y'x'} & \varepsilon_{y'y'} & \varepsilon_{y'z'} \\ \varepsilon_{z'x'} & \varepsilon_{z'y'} & \varepsilon_{z'z'} \end{pmatrix} \quad (1)$$

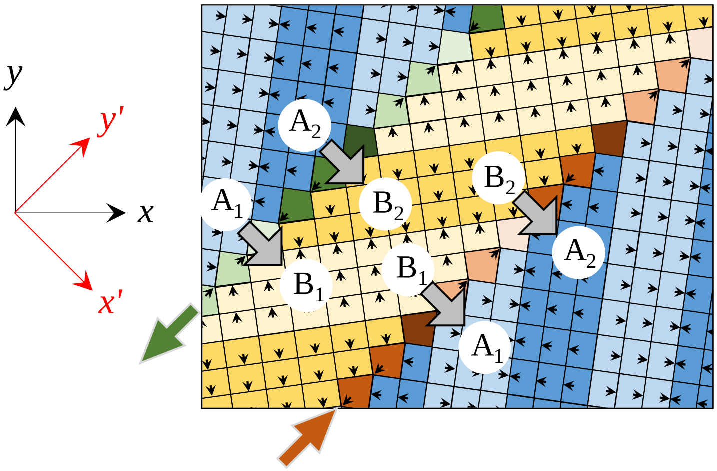

Supplementary Fig. 4 | Schematic of WO_3 ferroelastic domains. Four-variant monoclinic domains (A_1, A_2, B_1, and B_2) are shown with the color of bright blue, blue, bright yellow, and yellow respectively. Green and orange colored unit cells are strain-gradient-accumulated unit cells at ferroelastic domain walls. Small black arrows represent the directions of monoclinic shear deformation (see the Supplementary Fig. 5 for the details), and green and orange arrows represent the flexoelectric polarizations.

We set a cubic unit cell as a reference with the lattice parameter (l=3.71 Å), which is the average value of *a* and *b* parameters of monoclinic unit cell (a=3.66 Å, b=3.76 Å, c=3.85 Å). To express the strain state of monoclinic unit cell of WO_3, we use the strain constants of $\lambda = \left|\frac{a-l}{l}\right| = |0.05/3.71| \approx 0.013$, $\mu = |\tan 0.8°/2| \approx 0.007$, $\nu = \left|\frac{c-l}{l}\right| = |0.14/3.71| \approx 0.038$. Since A_1 and A_2 unit cells are compressed along *x*-axis and stretched along *y*-axis (Supplementary Fig. 5), ε_{xx} and ε_{yy} are represented as $-\lambda$ and λ, respectively. In a similar way, those of B_1 and B_2 unit cells in *x*-*y* plane are represented as λ and $-\lambda$. Monoclinic shear strains (ε_{xz} or ε_{yz}) and a longitudinal strain along *z*-axis (ε_{zz}) are expressed using μ and ν, respectively.

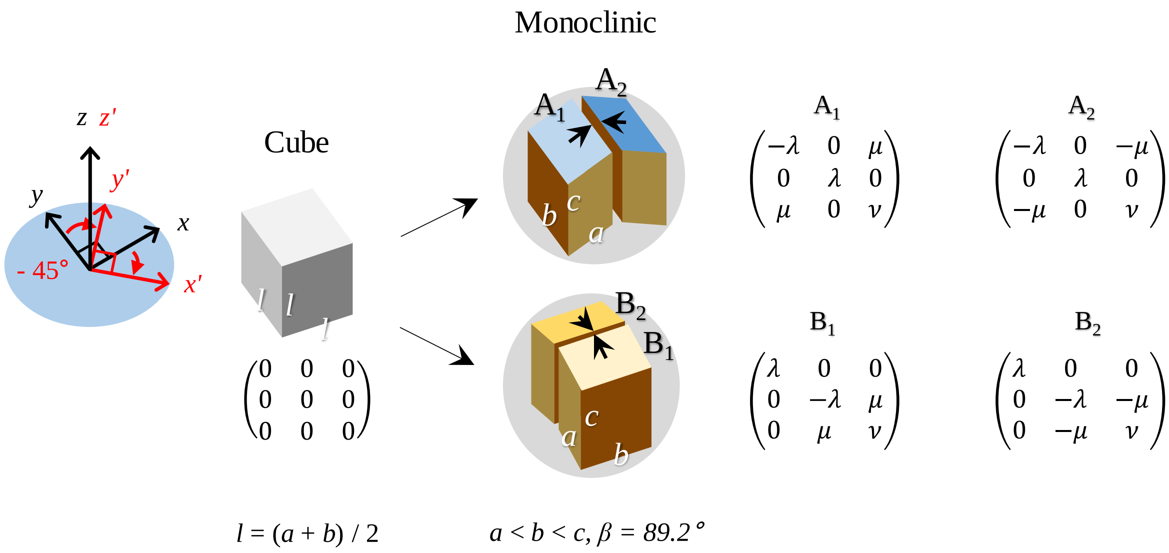

Supplementary Fig. 5 | Strain tensors of A_1, A_2, B_1, and B_2 monoclinic unit cells with the reference of cubic unit cell (gray cube). A_1, A_2, B_1, and B_2 monoclinic unit cells are colored with bright blue, blue, bright yellow, and yellow, respectively. Strain tensors for the unit cells are represented in the *xyz* coordinate axes.

It is convenient to represent the strain tensors in $x'y'z'$ coordinate system because the strain distribution has a translational symmetry along y' axis. Provided the basis axes are rotated around z axis (// z' axis) by θ, the conversion of six strain components can be expressed as,

$$\begin{pmatrix} \varepsilon_{x'x'} \\ \varepsilon_{y'y'} \\ \varepsilon_{z'z'} \\ \varepsilon_{y'z'} \\ \varepsilon_{x'z'} \\ \varepsilon_{x'y'} \end{pmatrix}_{\theta} = \begin{pmatrix} \cos^2\theta & \sin^2\theta & 0 & 0 & 0 & 2\sin\theta\cos\theta \\ \sin^2\theta & \cos^2\theta & 0 & 0 & 0 & -2\sin\theta\cos\theta \\ 0 & 0 & 1 & 0 & 0 & 0 \\ 0 & 0 & 0 & \cos\theta & \sin\theta & 0 \\ 0 & 0 & 0 & -\sin\theta & \cos\theta & 0 \\ -\sin\theta\cos\theta & \sin\theta\cos\theta & 0 & 0 & 0 & \cos^2\theta-\sin^2\theta \end{pmatrix} \begin{pmatrix} \varepsilon_{xx} \\ \varepsilon_{yy} \\ \varepsilon_{zz} \\ \varepsilon_{yz} \\ \varepsilon_{xz} \\ \varepsilon_{xy} \end{pmatrix}. \quad (2)$$

More specifically, the strain components of A_1 monoclinic unit cell in $x'y'z'$ coordinate system (*i.e.*, θ =45°) can be written as,

$$\begin{pmatrix} \varepsilon_{x'x'} \\ \varepsilon_{y'y'} \\ \varepsilon_{z'z'} \\ \varepsilon_{y'z'} \\ \varepsilon_{x'z'} \\ \varepsilon_{x'y'} \end{pmatrix}_{\theta=-45^\circ} = \begin{pmatrix} 1/2 & 1/2 & 0 & 0 & 0 & -1 \\ 1/2 & 1/2 & 0 & 0 & 0 & 1 \\ 0 & 0 & 1 & 0 & 0 & 0 \\ 0 & 0 & 0 & 1/\sqrt{2} & -1/\sqrt{2} & 0 \\ 0 & 0 & 0 & 1/\sqrt{2} & 1/\sqrt{2} & 0 \\ 1/2 & -1/2 & 0 & 0 & 0 & 0 \end{pmatrix} \begin{pmatrix} -\lambda \\ \lambda \\ \nu \\ 0 \\ \mu \\ 0 \end{pmatrix} = \begin{pmatrix} 0 \\ 0 \\ \nu \\ -\mu/\sqrt{2} \\ \mu/\sqrt{2} \\ -\lambda \end{pmatrix} \quad (3)$$

In a similar way, strain matrices for all the domains are obtained. We note all $\varepsilon_{x'x'}$ and $\varepsilon_{y'y'}$ components are zero.

$$\varepsilon_{\mathrm{A}_1} = \begin{pmatrix} 0 & -\lambda & \mu/\sqrt{2} \\ -\lambda & 0 & -\mu/\sqrt{2} \\ \mu/\sqrt{2} & -\mu/\sqrt{2} & \nu \end{pmatrix}, \quad \varepsilon_{\mathrm{A}_2} = \begin{pmatrix} 0 & -\lambda & -\mu/\sqrt{2} \\ -\lambda & 0 & \mu/\sqrt{2} \\ -\mu/\sqrt{2} & \mu/\sqrt{2} & \nu \end{pmatrix} \tag{4}$$

$$\varepsilon_{\mathrm{B}_1} = \begin{pmatrix} 0 & \lambda & \mu/\sqrt{2} \\ \lambda & 0 & \mu/\sqrt{2} \\ \mu/\sqrt{2} & \mu/\sqrt{2} & \nu \end{pmatrix}, \quad \varepsilon_{\mathrm{B}_2} = \begin{pmatrix} 0 & \lambda & -\mu/\sqrt{2} \\ \lambda & 0 & -\mu/\sqrt{2} \\ -\mu/\sqrt{2} & -\mu/\sqrt{2} & \nu \end{pmatrix} \tag{5}$$

Next, the strain difference tensors between two neighboring ferroelastic domains (*e.g.*, $\Delta\varepsilon_{\mathrm{A}_1\to\mathrm{B}_1} \equiv \varepsilon_{\mathrm{B}_1} - \varepsilon_{\mathrm{A}_1}$) are expressed as follows,

$$\Delta\varepsilon_{\mathrm{A}_1\to\mathrm{B}_1} = \begin{pmatrix} 0 & 2\lambda & 0 \\ 2\lambda & 0 & \sqrt{2}\mu \\ 0 & \sqrt{2}\mu & 0 \end{pmatrix}, \qquad \Delta\varepsilon_{\mathrm{A}_2\to\mathrm{B}_2} = \begin{pmatrix} 0 & 2\lambda & 0 \\ 2\lambda & 0 & -\sqrt{2}\mu \\ 0 & -\sqrt{2}\mu & 0 \end{pmatrix} \tag{6}$$

$$\Delta\varepsilon_{\mathrm{B}_1\to\mathrm{A}_1} = \begin{pmatrix} 0 & -2\lambda & 0 \\ -2\lambda & 0 & -\sqrt{2}\mu \\ 0 & -\sqrt{2}\mu & 0 \end{pmatrix}, \quad \Delta\varepsilon_{\mathrm{B}_2\to\mathrm{A}_2} = \begin{pmatrix} 0 & -2\lambda & 0 \\ -2\lambda & 0 & \sqrt{2}\mu \\ 0 & \sqrt{2}\mu & 0 \end{pmatrix} \tag{7}$$

The *z'*-axis related shear strain difference is as large as $\sqrt{2}\mu$ that is only ~2.6 times smaller than the in-plane shear strain difference 2*λ*. However, we ignore the effect of $\Delta\varepsilon_{y'z'}$ in the following discussion because fine-domain walls frequently emerges every a few nanometer along a given macro-domain wall and compensate their individual contributions each other. The non-canceling effects on flexoelectricity and flexopiezoelectricity over a macro-domain wall are obtained by averaging of $\Delta\varepsilon_{\mathrm{A}_1\to\mathrm{B}_1}$ and $\Delta\varepsilon_{\mathrm{A}_2\to\mathrm{B}_2}$ (or $\Delta\varepsilon_{\mathrm{B}_1\to\mathrm{A}_1}$ and $\Delta\varepsilon_{\mathrm{B}_2\to\mathrm{A}_2}$). Therefore, the strain difference tensors across the ferroelastic domain walls can be approximately written as,

$$\Delta\varepsilon_{\mathrm{A}\to\mathrm{B}} \approx \begin{pmatrix} 0 & 2\lambda & 0 \\ 2\lambda & 0 & 0 \\ 0 & 0 & 0 \end{pmatrix}, \qquad \Delta\varepsilon_{\mathrm{B}\to\mathrm{A}} \approx \begin{pmatrix} 0 & -2\lambda & 0 \\ -2\lambda & 0 & 0 \\ 0 & 0 & 0 \end{pmatrix}. \tag{8}$$

Each component of $\Delta\varepsilon_{B\rightarrow A}$ has opposite sign of that of $\Delta\varepsilon_{A\rightarrow B}$. Accordingly, only the in-plane shear components gradually change by the amount of 2λ (or -2λ) across the wall from A to B domain (or from B to A). This means that the shear strain gradient becomes positive or negative at ferroelastic domain walls, leading to the antiparallel flexoelectric polarizations. Such strain distribution in the vicinity of a wall can be imitated as a tangent hyperbolic function (tanh(x/w), where w is a half of the ferroelastic domain wall width)[2]. On the assumption of a ferroelastic domain wall width of ~20 nm, the maximal shear strain gradient at the center of a wall is estimated to be ~ 10^6 m^{-1} (Supplementary Fig.6).

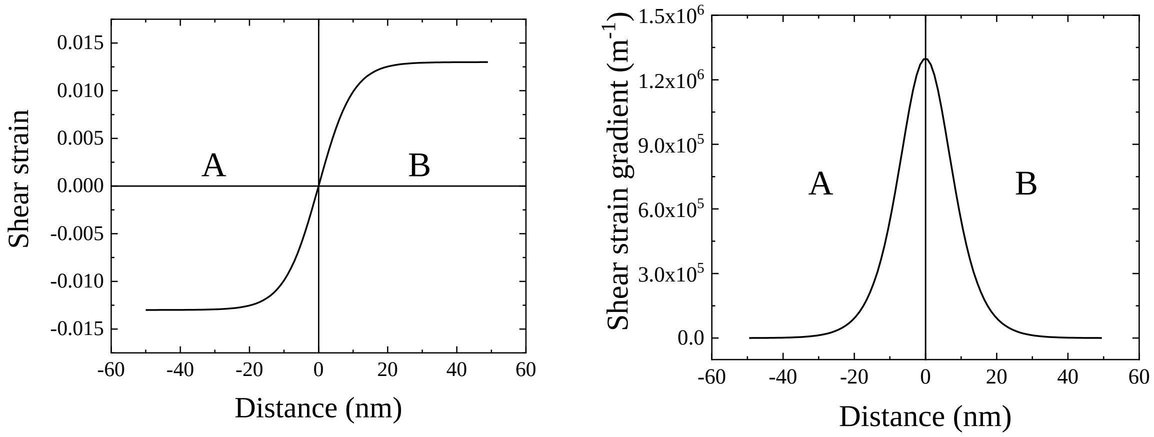

Supplementary Fig. 6 | Strain gradient across a ferroelastic domain wall. Distribution of tangent hyperbolic shear strain (left) and its derivative (strain gradient) across a ferroelastic domain wall (right).

Supplementary Note 3. PFM measurement and calibration of piezoelectric response

To compare the signals from macro- and fine-domain walls, we executed in-plane (IP) and out-of-plane (OOP) PFM measurements in the same area (Supplementary Fig. 7). As expected, OOP signals are much smaller than IP signals, even though OOP PFM measurement is much more sensitive. Accordingly, we could mainly focus on flexopiezoelectricity at macro-domain walls rather than fine-domain walls.

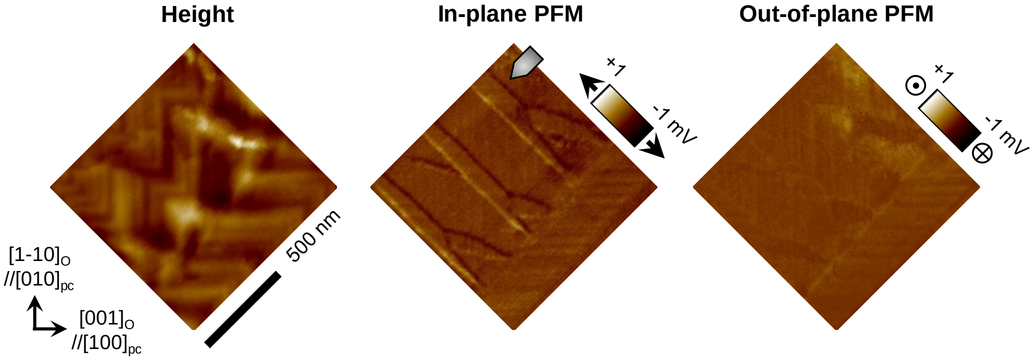

Supplementary Fig. 7 | In-plane and out-of-plane PFM images for the same area.

The torsional vibration of tip, that is induced by the lateral converse piezoelectric response, is sensed by the four-sector photodiode. For our PFM system, the surficial displacement per PFM signal was calibrated through finite element method (FEM) simulation and piezoelectric response measurement for a super-tetragonal $BiFeO_3$ thin film, as addressed in the paper of K. Chu *et al* [3]. The calibration factor of the displacement per PFM signal was 3.78 ± 1.12 pm per mV when amplification (×16) was used. Since our lateral PFM signal was measured to be ~0.4 mV without using the gain, the surficial displacement at ferroelastic domain walls recorded 12.1 pm (= 0.4 mV × 16 × 3.78 pm per mV × 0.5) in consideration of the spring constant of the cantilever, which is a half of the value used in the paper of K. Chu *et al* [3]. Furthermore, taking into account that an *ac* voltage of 2 V is applied to the PFM tip, the piezoelectric response can be determined to be ~6 pm/V.

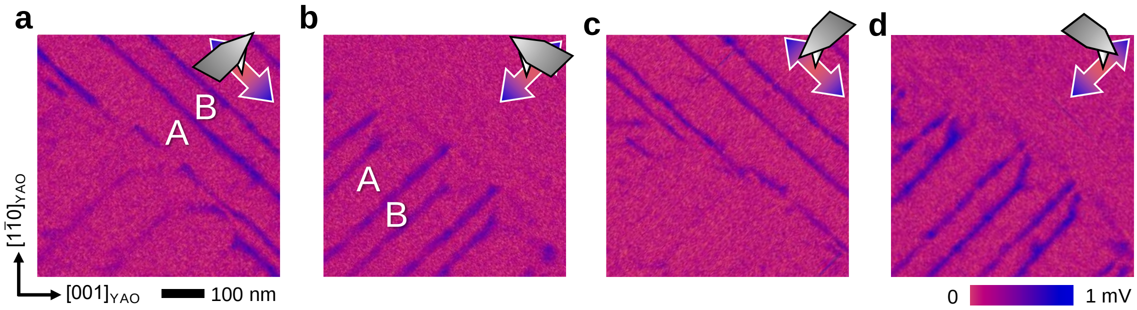

Supplementary Fig. 8 | Lateral piezoresponses at ferroelastic domain walls in a 200 nm-thick WO_3 film. Amplitude images for the four different cantilever orientations (from left to right, 45° (**a**), 135° (**b**), 225° (**c**), and 315° (**d**) from [001]$_{YAO}$). Blue color represents a larger piezoresponse signal compared to purple color (see the color scale of double arrow displayed with the tip schematic).

Supplementary Note 4. Phenomenological description of the flexopiezoelectricity

To explain the piezoresponse at ferroelastic domain walls, we introduce a trilinear term of strain (ε), strain gradient ($\partial\varepsilon/\partial x$), and polarization ($P$) with a sixth rank tensor of intrinsic flexopiezoelectric coefficient (θ^{f}) into the Landau free energy density (F):

$$\begin{aligned} F(P,\varepsilon) = F_0 + F_{\mathrm{elec}}(P) + F_{\mathrm{elas}}(\varepsilon) - E_i P_i - \sigma_{ij}\varepsilon_{ij} \\ -f_{ijkl}\frac{\partial\varepsilon_{kl}}{\partial x_j}P_i - \theta^{\mathrm{f}}_{ijklmn}\delta\varepsilon_{kl}\frac{\partial\varepsilon_{mn}}{\partial x_j}P_i, \end{aligned} \tag{9}$$

where, χ, C, E, σ, and f are the electric susceptibility (second rank), stiffness tensor (fourth rank), external electric field, external stress (second rank), and the flexoelectric coefficient tensor (fourth rank). The first term F_0 denotes the ground state energy density. As the spontaneous electric polarization of centrosymmetric materials is zero, the piezoelectric effect is not considered here. The electric energy density can be expressed as $F_{\mathrm{elec}} = \frac{1}{2\epsilon_0}\chi_{ij}^{-1}P_iP_j$, where ϵ_0 is the vacuum permittivity. A deviation of strain (ε_{ij}) from the eigenstrain (ε^0_{ij}) is represented by $\delta\varepsilon_{ij}(= \varepsilon_{ij} - \varepsilon^0_{ij})$. The elastic energy density can be written as a form of the Hook's law: $F_{\mathrm{elas}} = \frac{1}{2}C_{klmn}\delta\varepsilon_{kl}\delta\varepsilon_{mn}$ in the small deviation regime. The subscripts *i, j, k, l, m,* and *n* represent the indices of the spatial coordinate, *i.e.*, *x'*, *y'*, and *z'*. The Einstein convention that implies summation over repeating indices is used for simplicity. Using the energy minimization principle, the deviated strain can be written as,

$$\frac{\partial F}{\partial\varepsilon_{kl}} = \frac{\partial F_{\mathrm{elas}}}{\partial\varepsilon_{kl}} - \sigma_{kl} - \theta^{\mathrm{f}}_{ijklmn}\frac{\partial\varepsilon_{mn}}{\partial x_j}P_i = 0, \tag{10}$$

$$\delta\varepsilon_{ab} \approx C^{-1}_{abkl}\sigma_{kl} + C^{-1}_{abkl}\theta^{\mathrm{f}}_{ijklmn}\frac{\partial\varepsilon_{mn}}{\partial x_j}P_i. \tag{11}$$

Given external stress is zero, the strain change is mainly determined by the flexopiezoelectric term. Since electric polarization can be developed by external electric field with a correction of the flexoelectric effect:

$$\delta\varepsilon_{ab} \approx C^{-1}_{abkl}\theta^{\mathrm{f}}_{ijklmn}\frac{\partial\varepsilon_{mn}}{\partial x_j}\left[\epsilon_0\chi_{ip}E_p + \epsilon_0\chi_{ip}f_{pj'm'n'}\frac{\partial\varepsilon_{m'n'}}{\partial x_{j'}}\right]. \tag{12}$$

Therefore, the strain and polarization are given as,

$$\begin{aligned} \delta\varepsilon_{ab} &\approx C^{-1}_{abkl}\theta^{\mathrm{f}}_{ijklmn}\frac{\partial\varepsilon_{mn}}{\partial x_j}\epsilon_0\chi_{ip}E_p + C^{-1}_{abkl}\theta^{\mathrm{f}}_{ijklmn}\frac{\partial\varepsilon_{mn}}{\partial x_j}\epsilon_0\chi_{ip}f_{pj'm'n'}\frac{\partial\varepsilon_{m'n'}}{\partial x_{j'}} \\ &= d^{\mathrm{f}}_{abp}E_p + \varepsilon^{\mathrm{f}}_{ab}, \end{aligned} \tag{13}$$

$$P_i \approx \epsilon_0\chi_{ip}E_p + \epsilon_0\chi_{ip}f_{pj'm'n'}\frac{\partial\varepsilon_{m'n'}}{\partial x_{j'}} = \epsilon_0\chi_{ip}E_p + P^{\mathrm{f}}_i. \tag{14}$$

d^{f} stands for the converse piezoelectric coefficient tensor induced by strain gradient. P^{f} and ε^{f} are the flexoelectric polarization and its induced strain. When the electric field is zero, the induced strain and polarization are attributed only to the flexoelectricity. However, when the electric field is turned on, an additional strain emerges through the flexopiezoelectricity, which is induced by the trilinear term.

From the previous result of PFM data, the measured converse piezoelectric coefficient ($d^{\mathrm{f}}_{y'y'y'}$) was estimated as ~6 pm V^{-1}. Since, as in Eq. (13), this value is the multiplication of the elastic compliance ($C^{-1}_{y'y'y'y'}$~$1/Y$~ 4.1×10^{-12} m^2 N^{-1}, Y is the Young's modulus) (Ref.[4]), intrinsic flexopiezoelectric coefficient ($\theta^{\mathrm{f}}_{y'x'y'y'x'y'}$), strain gradient ($\partial\varepsilon_{x'y'}/\partial x'$ ~ 10^6 m^{-1}), electric permittivity in vacuum (~8.85×10^{-12} C V^{-1} m^{-1}), and the electric susceptibility of WO_3 ($\chi_{y'y'}$~ 5000) (Ref.[5]), the value of intrinsic flexopiezoelectric coefficient ($\theta^{\mathrm{f}}_{y'x'y'y'x'y'}$) is determined to be ~33 J C^{-1}.

Supplementary Note 5. Domain wall width and large-area strain by plane-view TEM analysis

Some of domain structures of the WO_3 thin film are sustained by the interaction with the film and substrate. Therefore, as the plane-view sample gets thinner, the domain structure gets relaxed due to the weaker clamping effect. For this reason, the plane-view TEM sample was prepared with the extreme care to maintain the domain structure using the very low energy. The mild condition of Ar^+ ion beam (1 kV, 1°) is applied to the 10 μm-thin foil sample. After the ion-milling process, the thinnest region of the sample is pierced by the beam, forming the thickness variation along the perimeter of the hole (Supplementary Figs. 9a and b). In Supplementary Fig. 9c, Area "(iii)", which is too thick to be transparent to the electron beam, exhibits the residual $YAlO_3$ substrate above the WO_3 film. On the other hand, the area "(i)" is too thin to see any domain structure because it is so away from the substrate that domain relaxation is easily made. Interestingly, the domain structure at the area "(ii)" is still visible, even though the region no longer has a substrate to support after polishing. The area "(ii)" is an optimal area which is thin enough to be capable of the STEM analysis on the domain structure. However, this area is distributed non-uniformly throughout the sample, so it is challenging to create an ideal area fitting to TEM/STEM imaging. FFT patterns of the areas "(i)" and "(ii)" also imply the same results (Supplementary Fig. 9c, d). We confirmed the intrinsic ferroelastic domain structure by SAED in the area "(ii)". The A and B ferroelastic domains can be transformed into each other through azimuthal rotation around the *c*-axis by ±90° (Supplementary Fig. 10).

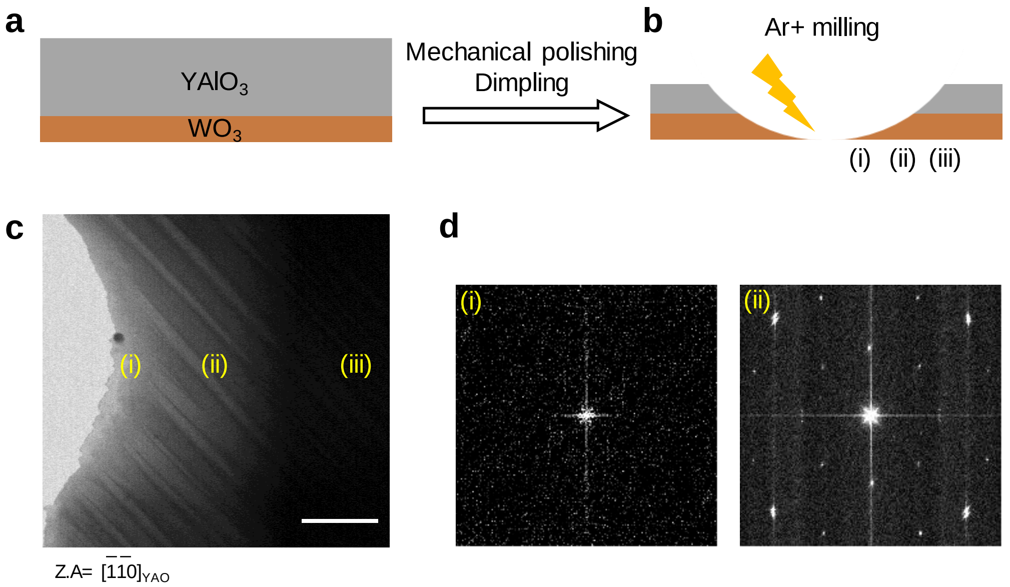

Supplementary Fig. 9 | Plane-view sample preparation and residual clamping effect. a, **b**, Schematic drawing of plane-view sample processing for WO_3 thin film. **c**, Low-magnification ABF-STEM image. Scale bar represents 500 nm. **d**, FFT patterns at the area of "(i)" and "(ii)". The single bright spot at the center in area "(i)" represents no periodic pattern meaning that the domain structures are disappeared, while the FFT pattern from area "(ii)" is indicative of the periodic patterns, formed by the domain structures.

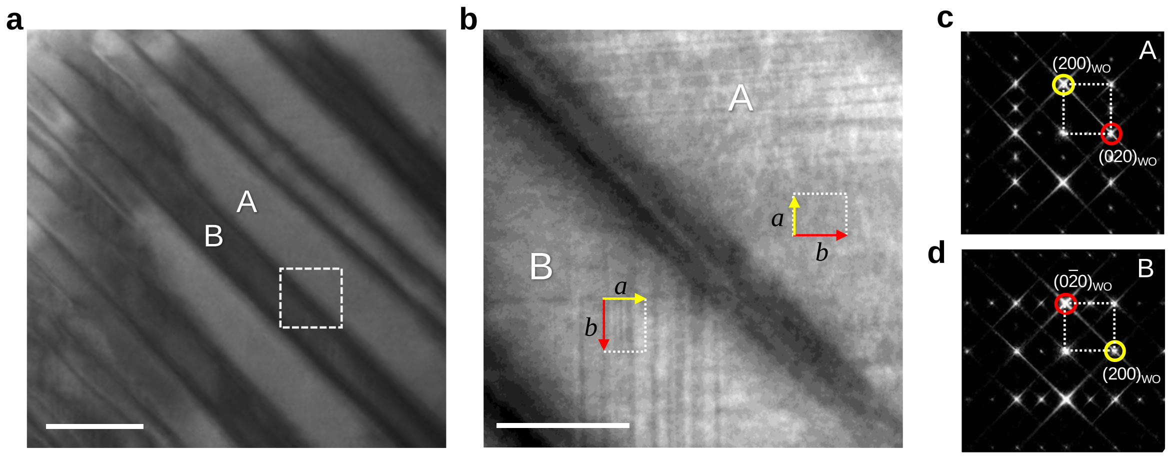

Supplementary Fig. 10 | Plane-view TEM analysis of WO_3 film. a, In-plane TEM image of a substrate-etched WO_3 thin film with a film thickness of ~300 nm. Zone axis is $[\bar{1}\bar{1}0]_{YAO}$. Scale bar represents 200 nm. **b**, Zoomed-in scanning TEM image shows a typical structure of A and B domains with -45° ferroelastic domain walls. Scale bar represents 50 nm. **c, d**, SAED patterns of A (c) and B (d) domains.

The sparse vertical or horizontal line contrast in the domain A and B in Fig. 3a most likely originates from the fine domain walls. However, the distance between the two lines doesn't represent the exact width of the fine domains, since the fine domains are considered to be partially relaxed. The fine domains are formed as a result of small mosaic rotations sharing the *a* or *b* axis, so they are easily relaxed into a single merged domain. Such weakness of the fine-domains was already observed in our previous study[6], revealing that the sizes of macro-domains and fine-domains depend on the film thickness and accordingly the strain field. In case of a 70 nm-thick WO_3 film, fine-domains are fragile; and they disappear after exposure to a weak electron beam. Due to its structural fragility, the ~300 nm-thick WO_3 film was fabricated in the current study. From the plane-view results of Supplementary Fig. 11, the macro- and fine-domains can be clearly identified and the width of fine-domains is measured to be ~10 nm, which is much smaller than that of the macro-domains.

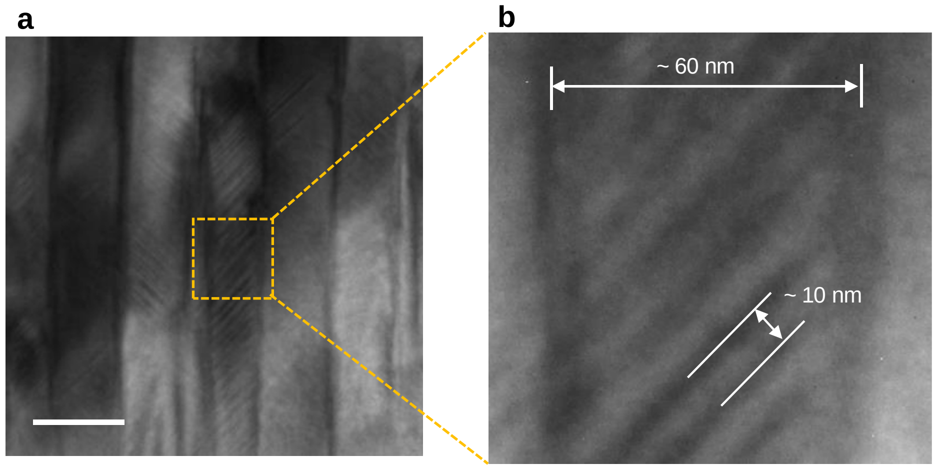

Supplementary Fig. 11 |Macro-domain walls and fine-domain walls from the plane-view TEM observation. a, Macro-domains and fine-domains are observed. Scale bar represents 100 nm. **b**, A magnification view of the area indicated by the yellow dashed box in **a**. The fine-domain walls with a width of ~10 nm periodically appear.

The domain wall width is directly measured with STEM images. In STEM images, the macro-domain walls appear with a brighter contrast due to the electron scattering at domain walls by irregular atomic arrays (atomic dechanneling). Supplementary Fig. 12 is an example figure for estimating the domain wall width. We took an area for making the average contrast profile across a macro-domain wall and measured the full-width-at-half-maximum (FWHM) of the Gaussian fit function. We similarly examined the FWHMs of 18 domain walls, and it was found that an average domain wall thickness is 17.85 nm with a standard deviation of 3.03 nm. The maximum and the minimum values were excluded.

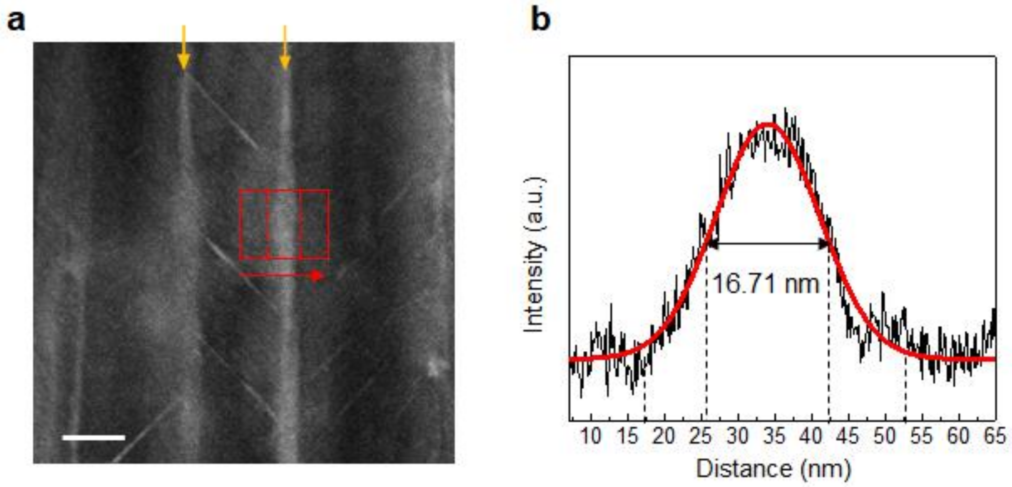

Supplementary Fig. 12 | Example of direct measurement of the domain wall width in STEM images. a, Domain wall from plane-view TEM observation. Two macro domain walls are indicated by yellow arrows. Scale bar represents 50 nm. **b,** Intensity profile of a selected area indicated by the red colored box in **a**. The measured domain wall width was approximately 16.71 nm.

For large-area strain analysis, we carried out the geometric phase analysis (GPA) analysis using plane-view low-magnified STEM image. The difference in strain between the two adjacent domains is expected to be ~3 %, however the lower value of ~1.5 % is detected in the cross sections of high-resolution strain maps (Fig. 3b, c). This is because the area in Fig. 3a is not sufficient to cover the full range of strain variations across the domains. Accordingly, analysis for full strain distribution over a larger area with containing a few domains is executed (Supplementary Fig. 13). As expected, strain differences of ~3 % between the domains are observed in the ε_{xx} and ε_{yy} maps. The observation suggests that a strain change of ~1.5 % occurs quickly within a small width (~15 nm) of domain walls and the remaining strain difference relaxes gradually over a larger width (up to ~50 nm, depending on domain width).

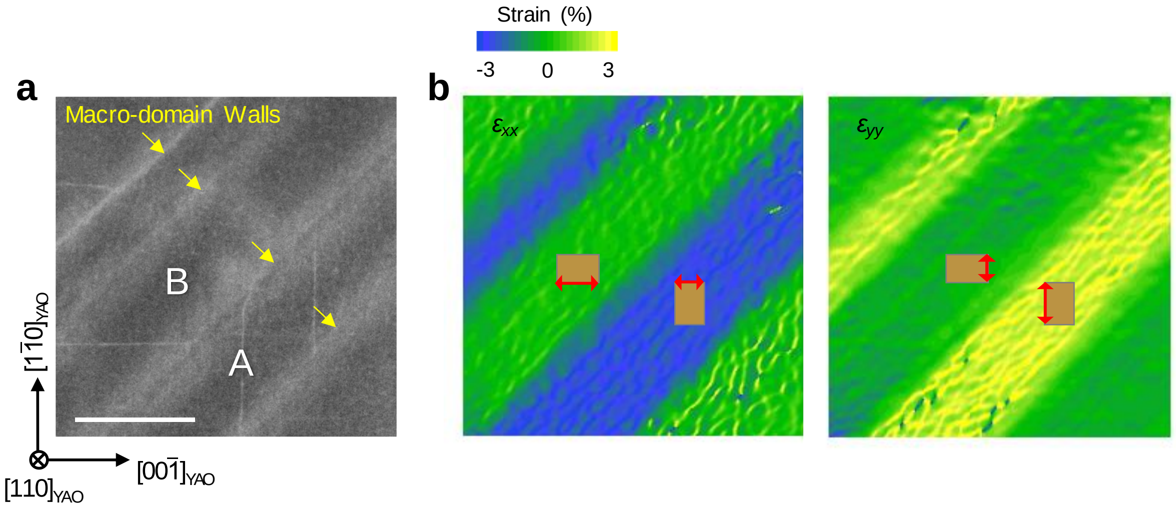

Supplementary Fig. 13 | Strain distribution over ferroelastic domains. a, Plane-view low-magnified STEM image of a ~300 nm-thick WO_3 film. Yellow arrows indicate the ferroelastic domain walls. Relatively bright contrast around the walls is observed, indicating strain-gradient regions. Scale bar represents 100 nm. **b,** Relative strain maps for the region of (a). Green areas are arbitrarily chosen as a zero strain reference. Blue and yellow regions relatively undergo −3 and +3 % strains compared to the green areas. A brown rectangle on each domain represents the orientation of pseudocubic unit cell. The red arrows represent the lattice parameters along x and y axes.

Supplementary Note 6. Strain gradient and domain wall width measured from X-ray diffraction

To support the existence of strain gradients at ferroelastic domain walls, we carried out x-ray diffraction to examine the diffusive nature of WO_3 $(001)_{pc}$ peak. Supplementary Fig. 14 is a quasi-HK reciprocal space map obtained by multiple ω-rocking scans at different azimuthal angles. From this map, we were able to identify the mosaic rotations of monoclinic unit cells by observing the $(001)_{pc}$ peaks on the H or K axes with a deviation from the center by the mosaic rotation angle. There are four kinds of monoclinic unit cell rotations in ferroelastic fine domains, which are labeled as A_1, A_2, B_1, and B_2. The shape of a peak has anisotropy in peak broadness, arising from the narrow width of a ferroelastic domain and the elongation along the other in-plane axis.

Indeed, we were able to observe the diffusive peaks. Besides, the diffusive feature between M peaks and A & B domain peaks suggests that there are structural gradients in the vicinity of macro-domain walls. To estimate the width of ferroelastic domain walls, the FWHM of M_4 peak was analyzed through deconvolution of peaks for a 45° magenta cross-sectional profile in Supplementary Fig. 14a (see the details in Supplementary Fig. 14b). Interestingly, the calculated wall width is ~26.5 nm (= ~3.71 Å / 0.014), which is consistent to the measured value (~20 nm) by the PFM and STEM. Thus, this non-destructive characterization enhances the credibility of the claim for gradual structural changes across the macro-domain walls.

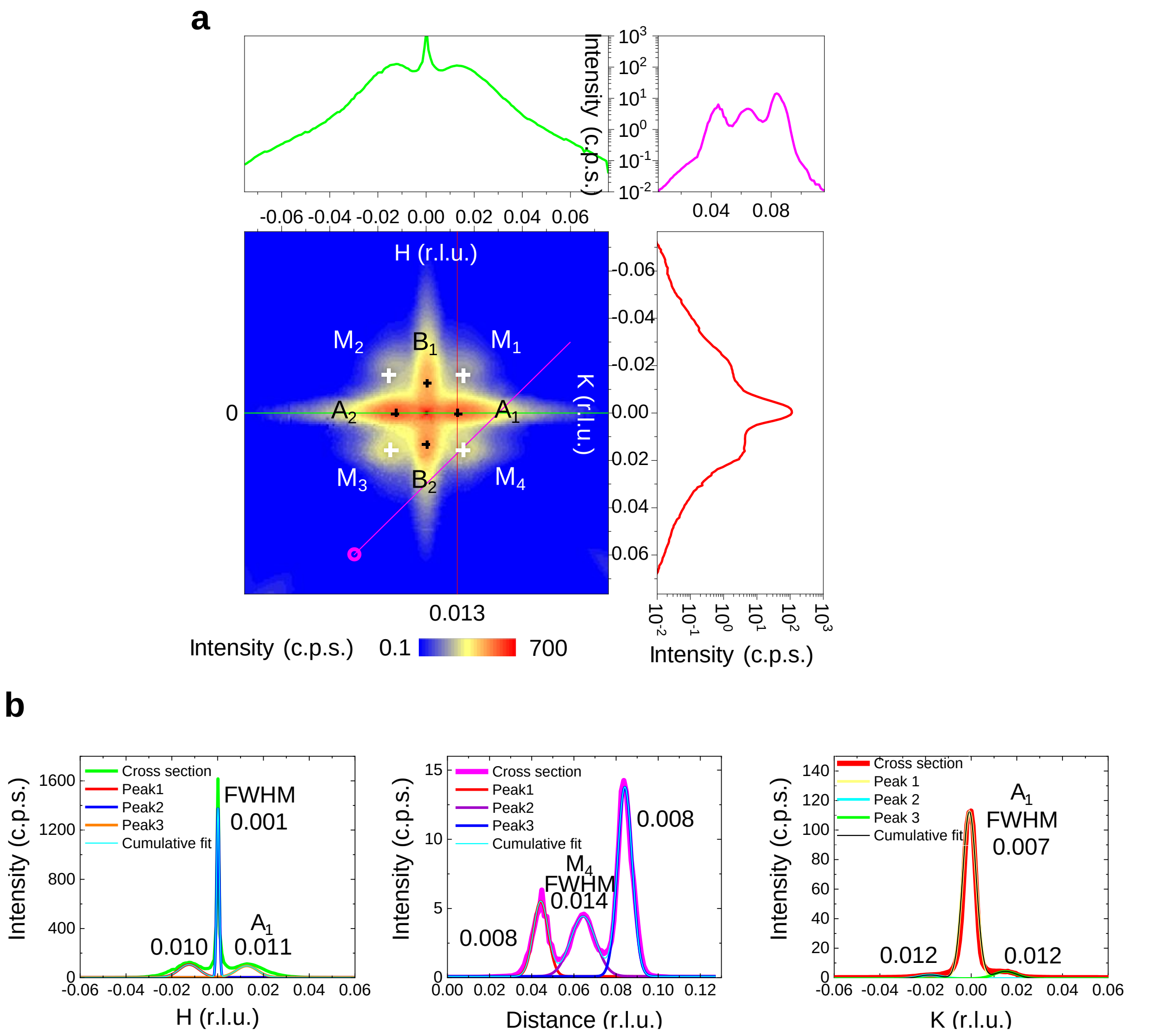

Supplementary Fig. 14 | X-ray diffraction of ferroelastic domains and walls. a, Semi-HK reciprocal space map at WO_3 (001), and its cross-sections for the ~420 nm-thick film. A_1, A_2, B_1, and B_2 represent the diffraction peaks of ferroelastic domains (fine-domains) with the different mosaic rotation angles. M_1, M_2, M_3, and M_4 indicate the intermediate phases at ferroelastic domain walls (macro-domain walls) which have a mosaic rotation angle of 45°, 135°, 225°, or 315°, respectively. Green, magenta, and red lines are cross-sections to obtain the FWHMs of A_1 and M_4 peaks. **b**, Deconvolution of the cross-sections. FWHMs for the deconvoluted peaks are indicated in reciprocal lattice unit (r.l.u.).

Supplementary Note 7. Flexoelectric polarizations at the center of ferroelastic domain wall

Flexoelectric polarization induced by strain-gradient is

$$P_i^{\mathrm{f}} = \sum_{jkl} \mu_{ijkl} \frac{\partial \varepsilon_{kl}}{\partial x_j} \tag{15}$$

where i, j, k, and l are x', y', or z'. P_i^{f} is the induced polarization in a direction of i, and $\partial \varepsilon_{kl}/\partial x_j$ is a strain gradient along j direction. μ_{ijkl} is the effective flexoelectric coefficient relating P_i^{f} to the strain gradient. Since we consider only shear strain components, $\partial \varepsilon_{x'y'}/\partial x'$(=$\partial \varepsilon_{y'x'}/\partial x'$), flexoelectric polarization terms are reduced to the three as follows.

$$P_{x'}^{\mathrm{f}} = \mu_{x'x'x'y'} \frac{\partial \varepsilon_{x'y'}}{\partial x'} + \mu_{x'x'y'x'} \frac{\partial \varepsilon_{y'x'}}{\partial x'} = 2\mu_{x'x'x'y'} \frac{\partial \varepsilon_{x'y'}}{\partial x'}, \tag{16}$$

$$P_{y'}^{\mathrm{f}} = \mu_{y'x'x'y'} \frac{\partial \varepsilon_{x'y'}}{\partial x'} + \mu_{y'x'y'x'} \frac{\partial \varepsilon_{y'x'}}{\partial x'} = 2\mu_{y'x'x'y'} \frac{\partial \varepsilon_{x'y'}}{\partial x'}, \tag{17}$$

$$P_{z'}^{\mathrm{f}} = \mu_{z'x'x'y'} \frac{\partial \varepsilon_{x'y'}}{\partial x'} + \mu_{z'x'y'x'} \frac{\partial \varepsilon_{y'x'}}{\partial x'} = 2\mu_{z'x'x'y'} \frac{\partial \varepsilon_{x'y'}}{\partial x'}. \tag{18}$$

To roughly estimate the flexoelectric effect, we use the following flexoelectric coefficients which are defined for isotropic dielectric materials[7],

$$\mu_{ijkl} = \frac{\alpha_1}{15}\left(\delta_{ik}\delta_{lj} + \delta_{il}\delta_{kj} + \delta_{ij}\delta_{kl}\right) + \frac{\alpha_2}{3}\left(\delta_{ik}\delta_{lj} + \delta_{il}\delta_{kj} - 2\delta_{ij}\delta_{kl}\right) \tag{19}$$

Based on this formula, $\mu_{x'x'x'y'}$ and $\mu_{z'x'x'y'}$ are zero. Therefore, only $P_{y'}^{\mathrm{f}}$ component is alive.

$$P_{x'}^{\mathrm{f}} = 0, \tag{20}$$

$$P_{y'}^{\mathrm{f}} = 2\mu_{y'x'x'y'} \frac{\partial \varepsilon_{x'y'}}{\partial x'}, \tag{21}$$

$$P_{z'}^{\mathrm{f}} = 0. \tag{22}$$

Qualitatively, the sign of $\mu_{y'x'x'y'}$ is negative[7],

$$\mu_{y'x'x'y'} = \frac{\alpha_1}{15}\left(\delta_{y'x'}\delta_{y'x'} + \delta_{y'y'}\delta_{x'x'} + \delta_{y'x'}\delta_{x'y'}\right) + \frac{\alpha_2}{3}\left(\delta_{y'x'}\delta_{y'x'} + \delta_{y'y'}\delta_{x'x'} - 2\delta_{y'x'}\delta_{x'y'}\right) = \frac{\alpha_1}{15} + \frac{\alpha_2}{3} < 0. \quad (23)$$

Therefore, the direction of induced flexoelectric polarizations are along *y'-axis* with the opposite sign of $\partial\varepsilon_{x'y'}/\partial x'$. Quantitatively, we can calculate the induced flexoelectric polarization. By assuming the shear flexoelectric coefficient as the multiplication of electric permittivity WO_3 (~$5000\epsilon_0$) and "flexovoltage" coefficient of one of perovskite oxides (−1.50 V) (Ref.[8]), the amount of flexoelectric polarization is calculated as ~13 μC cm^{-2}. ϵ_0 is the vacuum permittivity.

$$P^{\mathrm{f}}_{y'} \approx 2 \times (5000 \times 8.85 \times 10^{-12}\ \mathrm{C\ V^{-1}m^{-1}}) \times (-1.5\ \mathrm{V}) \times (10^{6}\ \mathrm{m^{-1}}) \approx -13.3\ \mathrm{\mu C\ cm^{-2}} \quad (24)$$

Supplementary Note 8. Phase field simulation

We performed a phase field simulation of a WO_3 system based on the Landau free energy density,

$$F(P,\varepsilon) = F_0 + F_{\mathrm{elec}}(P) + F_{\mathrm{elas}}(\varepsilon) - f_{ijkl}\frac{\partial \varepsilon_{kl}}{\partial x_j}P_i - \theta^{\mathrm{f}}_{ijklmn}\delta\varepsilon_{kl}\frac{\partial \varepsilon_{mn}}{\partial x_j}P_i + E_{\mathrm{G}}(\nabla\varepsilon). \qquad (25)$$

We set the zero-energy point at $P = 0$ and $\delta\varepsilon = 0$, i.e. $F_0 = 0$. The relative dielectric permittivity of the WO_3 is set to ~5,000 (Ref.[5]), so the electric energy is written as $F_{\mathrm{elec}}(P) = \frac{1}{2\epsilon_0}\chi_{ij}^{-1}P_iP_j = \frac{1}{10{,}000\epsilon_0}\left(P_x^2 + P_y^2 + P_z^2\right)$. Considering the spontaneous strain, the elastic energy can be written as $F_{\mathrm{elas}}(\varepsilon) = \frac{1}{2}C_{IJ}(\varepsilon_I - \varepsilon_I^0)(\varepsilon_J - \varepsilon_J^0)$. Here the capital indices *I*, *J* represent the Voigt notation, *i.e.* they run from 1 to 6 (1=*xx*, 2=*yy*, 3=*zz*, 4=*yz*, 5=*xz*, 6=*xy*). The spontaneous strain ε^0 for each of the four-variant fine-domains can be expressed as,

A1: $\varepsilon_1^{0(\mathrm{A1})} = -0.013,\ \varepsilon_2^{0(\mathrm{A1})} = 0.013,\ \varepsilon_3^{0(\mathrm{A1})} = 0.038,\ \varepsilon_4^{0(\mathrm{A1})} = 0,\ \varepsilon_5^{0(\mathrm{A1})} = 0.014,\ \varepsilon_6^{0(\mathrm{A1})} = 0;$
A2: $\varepsilon_1^{0(\mathrm{A2})} = -0.013,\ \varepsilon_2^{0(\mathrm{A2})} = 0.013,\ \varepsilon_3^{0(\mathrm{A2})} = 0.038,\ \varepsilon_4^{0(\mathrm{A2})} = 0,\ \varepsilon_5^{0(\mathrm{A2})} = -0.014,\ \varepsilon_6^{0(\mathrm{A2})} = 0;$
B1: $\varepsilon_1^{0(\mathrm{B1})} = 0.013,\ \varepsilon_2^{0(\mathrm{B1})} = -0.013,\ \varepsilon_3^{0(\mathrm{B1})} = 0.038,\ \varepsilon_4^{0(\mathrm{B1})} = 0.014,\ \varepsilon_5^{0(\mathrm{B1})} = 0,\ \varepsilon_6^{0(\mathrm{B1})} = 0;$
B2: $\varepsilon_1^{0(\mathrm{B2})} = 0.013,\ \varepsilon_2^{0(\mathrm{B2})} = -0.013,\ \varepsilon_3^{0(\mathrm{B2})} = 0.038,\ \varepsilon_4^{0(\mathrm{B2})} = -0.014,\ \varepsilon_5^{0(\mathrm{B2})} = 0,\ \varepsilon_6^{0(\mathrm{B2})} = 0.$

The elastic tensor C_{IJ} is constructed based on the Young's modulus (*Y*) and Poisson's ratio (γ) assuming a spherical symmetry *i.e.* $C_{11} = C_{22} = C_{33} = \frac{1-\gamma}{(1+\gamma)(1-2\gamma)}Y$, $C_{12} = C_{21} = C_{23} = C_{32} = C_{31} = C_{13} = \frac{\gamma}{(1+\gamma)(1-2\gamma)}Y$, $C_{44} = C_{55} = C_{66} = \frac{1}{1+\gamma}Y$, and otherwise 0. The Young's modulus and Poisson's ratio of the WO_3 are 243.41 GPa and 0.25297 (Ref.[4]).

To cope with the lack of the detailed knowledge of the flexoelectric and flexopiezoelectric tensors, we assume that they are isotropic and we thus truncate the contributions related to the high order spherical harmonics. By this means, the tensor components can be determined, vastly reducing the complexity of the high-order tensors. For the flexoelectric tensor, it is assumed that the flexoelectric effect induced by the longitudinal strain gradient (*e.g.* $\frac{\partial \varepsilon_{xx}}{\partial x}$) is much smaller than the one induced by the transverse strain gradient (*e.g.* $\frac{\partial \varepsilon_{yy}}{\partial x}$). Satisfying $f_{xxyy} = 1.5$ [V], the other coefficients can be found through the relation:

$$\mu_{ijkl} = -1.5\ [\mathrm{V}] \times \frac{1}{2}\left(\delta_{ik}\delta_{lj} + \delta_{il}\delta_{kj} - 2\delta_{ij}\delta_{kl}\right). \qquad (26)$$

Similarly, the flexopiezoelectric tensor's coefficient $\theta^{\mathrm{f}}_{xyxxyx} = 33$ [J/C] and the other coefficients are set so as to satisfy the spherical symmetry condition. The flexopiezoelectric tensor explicitly has the following form (where 1, 2, 3 stand for the spatial axes, *x*, *y*, *z*):

$$\theta^{\mathrm{f}}_{ijklmn} = 33\ \ [\mathrm{J/C}] \times \frac{1}{15}\mathrm{A}_{ijklmn}, \tag{27}$$

where A_{ijklmn} is an isotropic dimensionless tensor of rank 6, as listed in the Table S1.

A	…111	…211	…311	…121	…221	…321	…131	…231	…331	…112	…212	…312	…122	…222	…322	…132	…232	…332	…113	…213	…313	…123	…223	…323	…133	…233	…333
111…	12	0	0	0	1	0	0	0	1	0	1	0	-6	0	0	0	0	0	0	0	1	0	0	0	-6	0	0
211…	0	8	0	1	0	0	0	0	0	1	0	0	0	-6	0	0	0	-2	0	0	0	0	0	-2	0	-2	0
311…	0	0	8	0	0	0	1	0	0	0	0	0	0	0	-2	0	-2	0	1	0	0	0	-2	0	0	0	-6
121…	0	-6	0	15	0	0	0	0	0	15	0	0	0	8	0	0	0	5	0	0	0	0	0	5	0	-2	0
221…	-6	0	0	0	1	0	0	0	-2	0	1	0	8	0	0	0	0	0	0	0	-2	0	0	0	-2	0	0
321…	0	0	0	0	0	5	0	-2	0	0	0	5	0	0	0	5	0	0	0	-2	0	5	0	0	0	0	0
131…	0	0	-6	0	0	0	15	0	0	0	0	0	0	0	-2	0	5	0	15	0	0	0	5	0	0	0	8
231…	0	0	0	0	0	-2	0	5	0	0	0	-2	0	0	0	5	0	0	0	5	0	5	0	0	0	0	0
331…	-6	0	0	0	-2	0	0	0	1	0	-2	0	-2	0	0	0	0	0	0	0	1	0	0	0	8	0	0
112…	0	8	0	1	0	0	0	0	0	1	0	0	0	-6	0	0	0	-2	0	0	0	0	0	-2	0	-2	0
212…	8	0	0	0	15	0	0	0	5	0	**15**	0	-6	0	0	0	0	0	0	0	5	0	0	0	-2	0	0
312…	0	0	0	0	0	5	0	5	0	0	0	5	0	0	0	-2	0	0	0	5	0	-2	0	0	0	0	0
122…	-6	0	0	0	1	0	0	0	-2	0	1	0	8	0	0	0	0	0	0	0	-2	0	0	0	-2	0	0
222…	0	-6	0	1	0	0	0	0	0	1	0	0	0	12	0	0	0	1	0	0	0	0	0	1	0	-6	0
322…	0	0	-2	0	0	0	-2	0	0	0	0	0	0	0	8	0	1	0	-2	0	0	0	1	0	0	0	-6
132…	0	0	0	0	0	-2	0	5	0	0	0	-2	0	0	0	5	0	0	0	5	0	5	0	0	0	0	0
232…	0	0	-2	0	0	0	5	0	0	0	0	0	0	0	-6	0	15	0	5	0	0	0	15	0	0	0	8
332…	0	-2	0	-2	0	0	0	0	0	-2	0	0	0	-6	0	0	0	1	0	0	0	0	0	1	0	8	0
113…	0	0	8	0	0	0	1	0	0	0	0	0	0	0	-2	0	-2	0	1	0	0	0	-2	0	0	0	-6
213…	0	0	0	0	0	5	0	5	0	0	0	5	0	0	0	-2	0	0	0	5	0	-2	0	0	0	0	0
313…	8	0	0	0	5	0	0	0	15	0	5	0	-2	0	0	0	0	0	0	0	15	0	0	0	-6	0	0
123…	0	0	0	0	0	5	0	-2	0	0	0	5	0	0	0	5	0	0	0	-2	0	5	0	0	0	0	0
223…	0	0	-2	0	0	0	-2	0	0	0	0	0	0	0	8	0	1	0	-2	0	0	0	1	0	0	0	-6
323…	0	-2	0	5	0	0	0	0	0	5	0	0	0	8	0	0	0	15	0	0	0	0	0	15	0	-6	0
133…	-6	0	0	0	-2	0	0	0	1	0	-2	0	-2	0	0	0	0	0	0	0	1	0	0	0	8	0	0
233…	0	-2	0	-2	0	0	0	0	0	-2	0	0	0	-6	0	0	0	1	0	0	0	0	0	1	0	8	0
333…	0	0	-6	0	0	0	1	0	0	0	0	0	0	0	-6	0	1	0	1	0	0	0	1	0	0	0	12

Supplementary Table 1 | The tensor element of isotropic $\mathbf{A}_{ijklmn}$.

The Ginzburg (strain gradient) energy E_{G} is modeled as

$$E_{\mathrm{G}} = G_{\mathrm{M}}\left(\frac{\partial\varepsilon_1}{\partial x}\right)^2 + G_{\mathrm{M}}\left(\frac{\partial\varepsilon_2}{\partial x}\right)^2 + G_{\mathrm{M}}\left(\frac{\partial\varepsilon_3}{\partial x}\right)^2 + G_{\mathrm{M}}\left(\frac{\partial\varepsilon_4}{\partial x}\right)^2 + G_{\mathrm{F}}\left(\frac{\partial\varepsilon_5}{\partial x}\right)^2 + \frac{1}{2}G_{\mathrm{M}}\left(\frac{\partial\varepsilon_6}{\partial x}\right)^2 + G_{\mathrm{M}}\left(\frac{\partial\varepsilon_1}{\partial y}\right)^2 + G_{\mathrm{M}}\left(\frac{\partial\varepsilon_2}{\partial y}\right)^2 + G_{\mathrm{M}}\left(\frac{\partial\varepsilon_3}{\partial y}\right)^2 + G_{\mathrm{F}}\left(\frac{\partial\varepsilon_4}{\partial y}\right)^2 + G_{\mathrm{M}}\left(\frac{\partial\varepsilon_5}{\partial y}\right)^2 + \frac{1}{2}G_{\mathrm{M}}\left(\frac{\partial\varepsilon_6}{\partial y}\right)^2, \tag{28}$$

where G_{M}=1.0×10^{-5} [J m^{-1}] and G_{F} =1.0×10^{-8} [J m^{-1}]. G_{F} was set intentionally to a small value to get a sharp interface at the fine-domain walls. Our system was reduced into a two-dimensional model assuming the layer was identical along the thickness direction, and the periodic boundary condition was applied along the in-plane directions. The spatial sampling rate was set to be 5 nm per pixel and the pixel grid size was 80-by-80, so the resultant dimension of the model space was 400×400×5 nm^3. The Metropolis algorithm was employed for the convergence.

We can successfully stabilize the hierarchical domain structure consisting of fine-domains and macro-domains as shown in Supplementary Figs. 15−17. The visualization of the strains, strain gradients, and the electric polarizations over the domains and across the domain walls provides useful insight into the twin structure. The macro-domain walls have a wide width of ~40 nm. The emergence of the flexoelectric polarizations at the macro-domain walls is also

reproduced. The magnitude of the in-plane polarizations is ~8 μC cm^{-2} (Supplementary Fig. 17b), which is of the same order of magnitude as the analytically estimated value. From the phase field simulation, we can not only verify the experimental observations, but also extract the additional features we didn't pay attention to before. A significant value of P_z is observed at fine domain walls due to the large shear strain gradient across the thin walls. In reality, two neighboring fine-domains face each other with a sharp interface. It brings us an open question whether the Landau theory is still valid for describing discontinuities or abrupt jumps in the order parameter field at the atomic scale where the continuum approximation is failed.

Another interesting point is that the hierarchical twin structure can be stabilized without the substrate constraint. The structure itself is in a meta-stable state. We can interpret that the misfit strain from substrate is used for selection of the structure among various possible candidates. Proper matching of the lattice parameters between the structure and the substrate manifests itself as a hierarchical twin structure with minimal deformation. So, we can understand that some regions of a TEM specimen for the planar view still endure the relaxation.

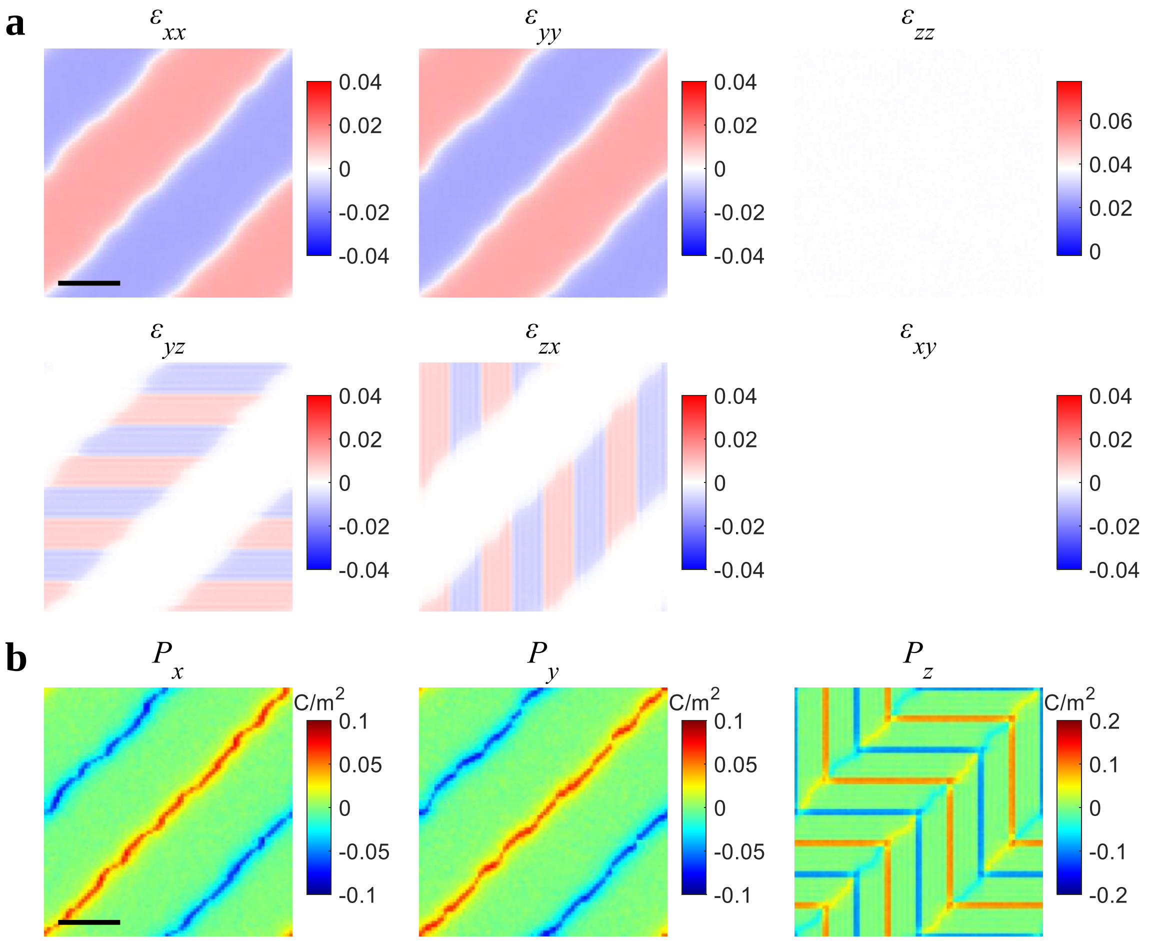

Supplementary Fig. 15 | Simulation result of the strain and polarization fields. a, Spatial distribution of strains. **b,** Spatial distribution of ferroelectric polarization. The *x* and *y* axes are parallel to the edges of the plot and the *z*-axis is out of the plane. Scale bars indicate 100 nm.

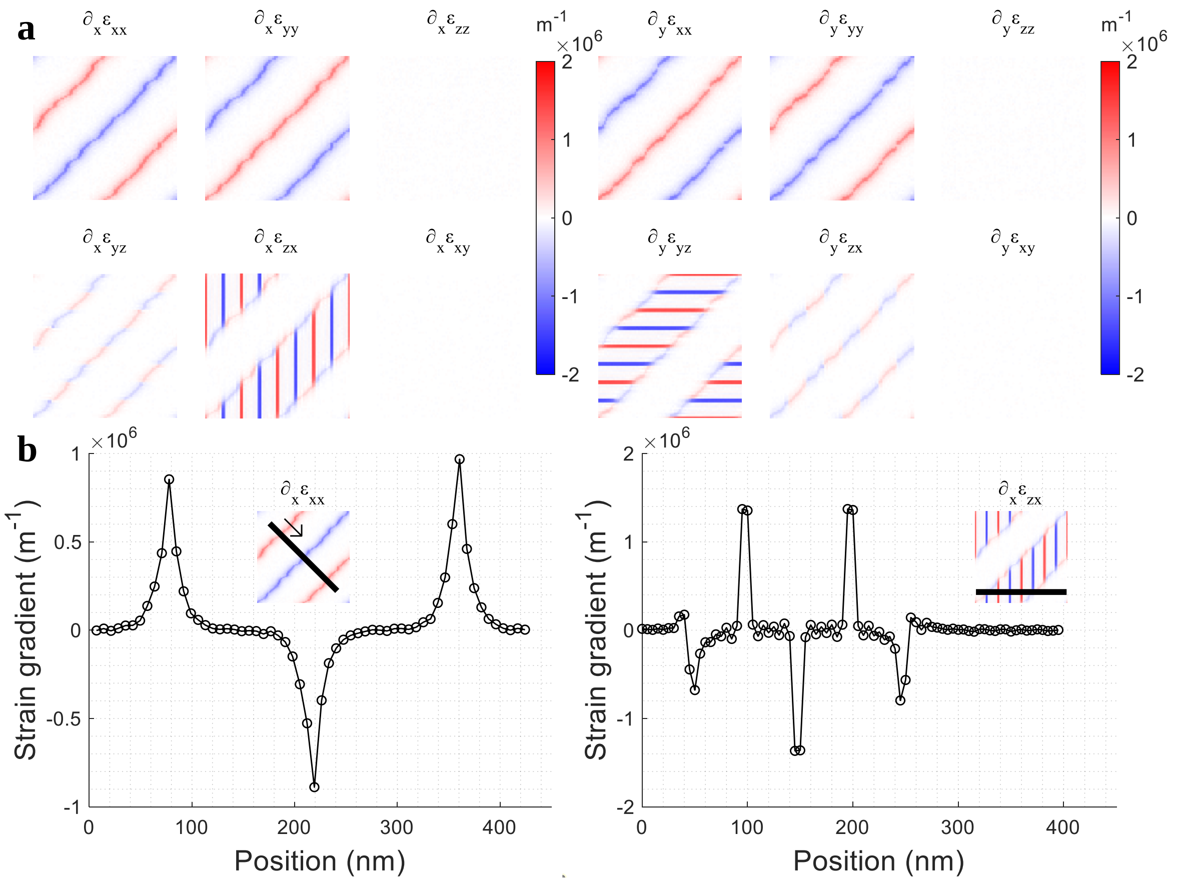

Supplementary Fig. 16 | Strain gradient maps and cross-sectional profiles. a, Strain gradient maps. The *z*-axis derivatives are all zero so the corresponding images are omitted. The *x* and *y* axes are parallel to the edges of the plot and the *z*-axis is out of the plane. **b,** Line profiles of two chosen strain gradients, which show gradual and stiff changes of strains at the macro-domain walls (left) and fine-domain walls (right), respectively.

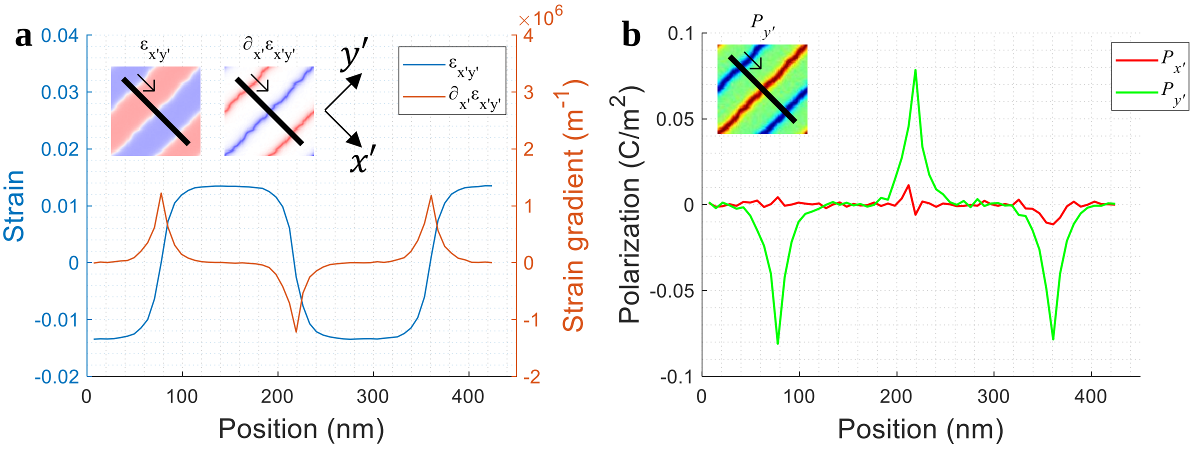

Supplementary Fig. 17 | Line profiles of strain and polarization in the *x*′*y*′*z*′ Cartesian coordinate. a, Line profiles of strain and strain gradient along *x*′-axis. **b**, Line profiles of ferroelectric polarization components which are parallel (*y*′) or perpendicular (*x*′) to domain walls within the surface.

Supplementary Note 9. Detection of depolarized fields using DPC-STEM

To directly measure the depolarization field, we carried out the DPC-STEM imaging for plane-view and cross-sectional TEM samples. DPC STEM is very sensitive to strain, electric field, thickness variation, and diffraction condition. Firstly, to reduce the experimental error caused by local thickness variations of a TEM sample, TEM samples was fabricated by FIB. The FIBed sample is relatively thick so that strain can maintain and the film thickness variation can be minimized.

The schematic illustration of the 8 segmented detectors used for this study is shown in Supplementary Fig. 18a, wherein the yellow disk indicates a bright-field beam. The tilting of a TEM specimen is crucial in DPC STEM imaging to avoid dynamical diffraction effects. We performed the DPC STEM analysis by tilting a sample far away from zone-axis to minimize effect of diffraction contrast. However, the tilting range was limited because our WO_3 film structure is complicating, in which twin domains are rotated by ±45° relative to $[1\bar{1}0]_{YAO}$ or $[001]_{YAO}$ zone axes. Therefore, to avoid the diffraction contrast, we obtained DPC images under two different conditions; at the zone-axis condition and off the zone-axis conditions. Supplementary Fig. 18b shows the cross-sectional low-magnification BF-TEM image of $WO_3/YAlO_3$ sample at the $[1\bar{1}0]_{YAO}$ zone-axis condition, where the bright and dark diffraction contrasts correspond to A and B domains, respectively. The intensity of DPC STEM image arises from the beam deflection, which is proportional to the magnitude of the electric field[9, 10]. If the detector segments 5-8 of layer 2 are used for electric field mapping and their orientations are aligned as shown in Supplementary Fig. 18a, the total beam deflection along the x and y direction can be calculated by using the simple relationship as follows.

$$d_x = (5+8)-(6+7)$$
$$d_y = (7+8)-(5+6) \quad (29)$$

We present the simultaneously-obtained DPC STEM images from detector segments 5-8 and the differential DPC-STEM images corresponding to d_x and d_y (Supplementary Fig. 18c-e). The (5+8)−(6+7) DPC-STEM image clearly shows contrast variation across the domain B, while the (7+8)−(5+6) image shows no significant image contrast within the WO_3 film. It means that there are relatively strong electric fields along the in-plane direction. To visualize accurate electric field components (E_x and E_y), we measured the angle between the x direction of segmented detector and the beam deflection direction, and then calibrated the results, as shown in Supplementary Fig. 19. Unlike the diffraction contrast shown in the BF-TEM image of Supplementary Fig. 19a, contrast inversion was observed within B domains in the $E_{[001]YAO}$ electric-field map (Supplementary Fig. 19c).

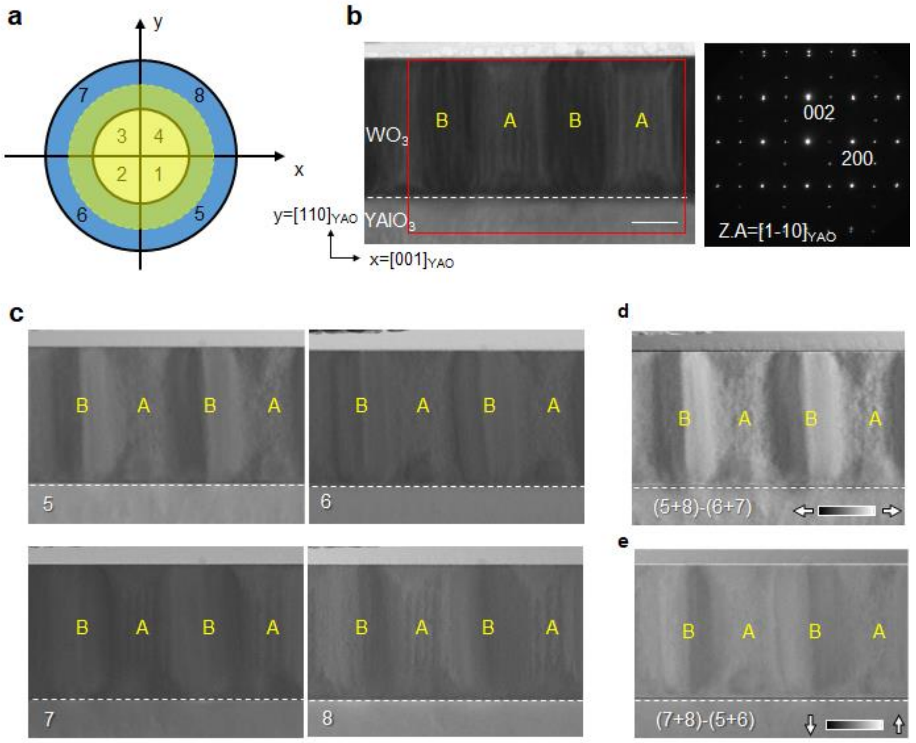

Supplementary Fig. 18 | DPC-STEM images simultaneously observed by the segmented detector. a, Schematic illustration of the segmented detector used for this study. **b**, BF-TEM image and the corresponding electron diffraction pattern taken along the $[1\bar{1}0]_{\mathrm{YAO}}$ zone-axis. Scale bar represents 100 nm. **c**, DPC-STEM images formed by detector segments 5-8 schematically illustrated in **a**. **d**, **e**, DPC-STEM images obtained by calculation according to the orientation relation between sample and detector.

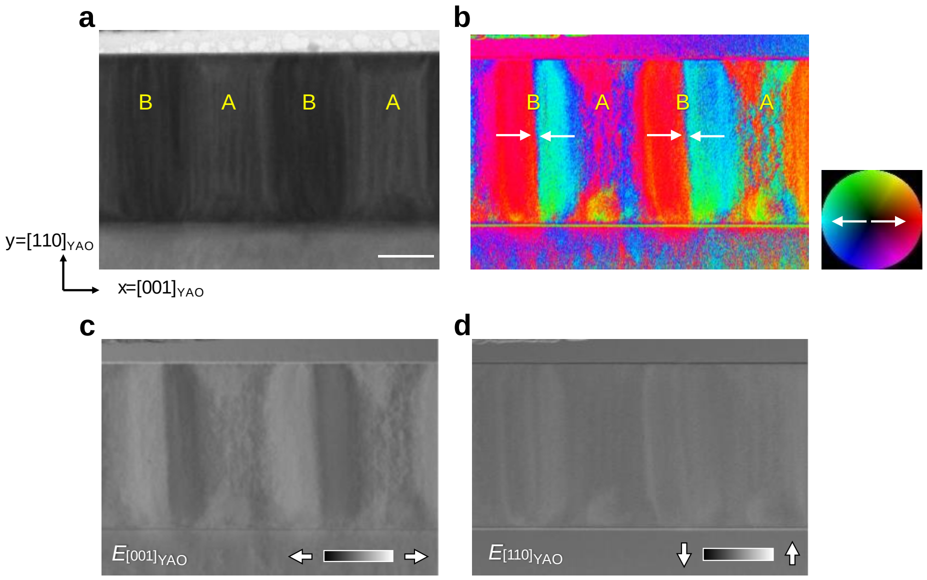

Supplementary Fig. 19 | Projected electric field vector map using DPC-STEM at the zone axis condition. **a**, BF-TEM image taken along the $[1\bar{1}0]_{\mathrm{YAO}}$ zone-axis. Scale bar represents 100 nm. **b**, Projected electric field vector map. **c**, **d**, E_x and E_y maps obtained from the segmented detectors. Electric field maps along x and y direction were calibrated considering the angle between the x direction of segmented detector and the beam deflection direction.

In order to check the effect of diffraction condition, we carried out the similar DPC experiments at different diffraction conditions. Supplementary Fig. 20 shows a DPC-STEM result taken at a condition slightly tilted away from the zone-axis. Although the contrast in BF-TEM image was changed by the diffraction condition, the electric field map along the *x* direction is almost the same as the result in Supplementary Fig. 19. This clarifies that the contrast inversion is not caused by diffraction contrast effect in WO_3 film.

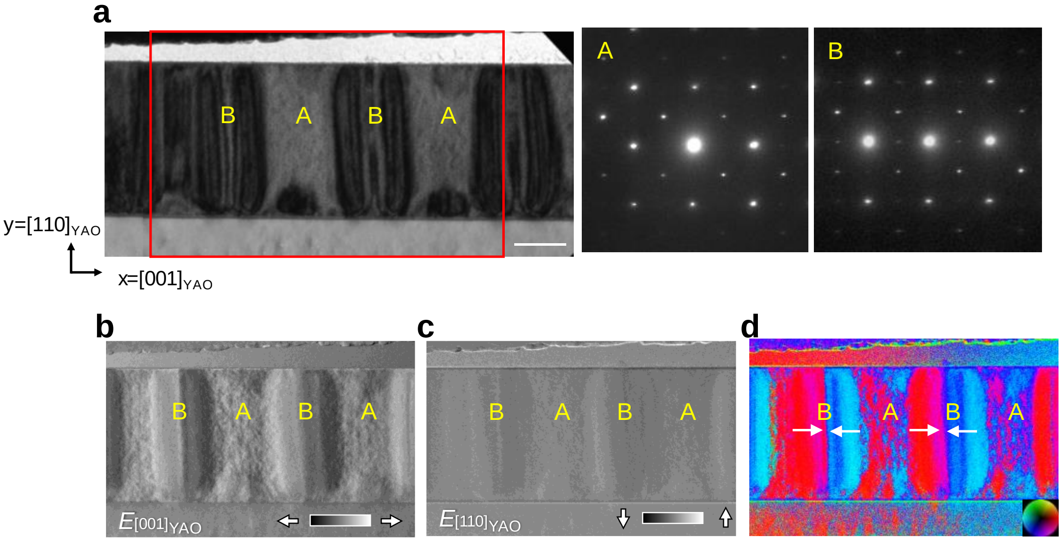

Supplementary Fig. 20 | Projected electric field vector map obtained off the zone-axis. a, BF-TEM image and the corresponding diffraction patterns of domain A and B. Sample is slightly tilted away from the $[1\bar{1}0]_{YAO}$ zone-axis. Scale bar represents 100 nm. **b**, **c**, E_x and E_y maps obtained from the segmented detectors. **d**, Projected electric-field vector map. Electric field map was calibrated considering the angle between the *x* direction of segmented detector and the beam deflection direction.

It is difficult to obtain more intuitive DPC-STEM data beyond the results shown in Supplementary Figs. 18-20, because the twin domain walls of WO_3 were inclined 45° with respect to the viewing directions and therefore the domain walls were not sharp, mixed up with two domains. In addition, the intensity signal in the DPC data can be attributed to strain distribution within the WO_3 film.

We additionally prepared a TEM specimen for a diagonal cut so that the twin domain walls are sharp, right oriented along a viewing direction. The depolarized electric fields in this geometry are oriented along the viewing direction, and so no DPC contrast is anticipated. The strain distribution across the twin domain wall in WO_3 were observed by atomic STEM imaging (similarly as presented in Fig. 3 of the main manuscript). However, any significant DPC contrast, that could arise from non-uniform strains in the DPC-STEM images, was not observed (The signal was only 2 times larger than our noise level evaluated in vacuum; Supplementary Fig. 21). This result shows good agreement with our expectation of the depolarized fields. Although the strain and diffraction contrasts may be included in our results, it is clear that the effects are not considerable. Accordingly, our comprehensive interpretation

are led to the fact that the significantly large (32 times larger than the noise level) signals in the DPC-STEM data along $[1\bar{1}0]_{YAO}$ zone-axis are mainly due to the electric fields within WO_3 film rather than the other effects.

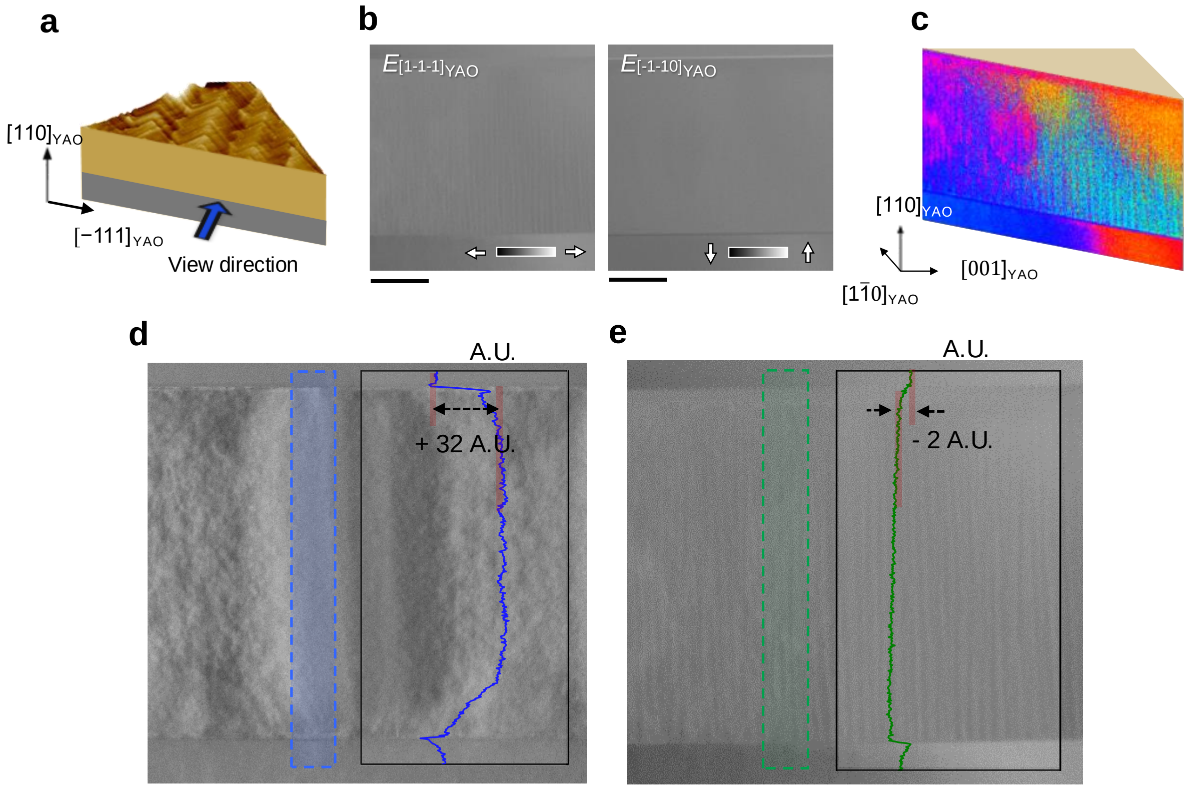

Supplementary Fig. 21 | Electric fields map by DPC-STEM for a diagonal cut, and quantitative comparison of signal strengths. a, Schematic view of an experimental geometry. **b**, DPC-STEM images taken for a diagonal cut surface with $[\bar{1}1\bar{1}]_{YAO}$ zone axis. The twin walls of WO_3 film are oriented in a direction parallel to the viewing direction. **b**, Projected electric field maps along the horizontal and vertical directions. Scale bars represent 100 nm. **c**, Constructed electric field vector map. **d** and **e**, Intensity profiles of E_x of Supplementary Fig. 20 and **b**. Intensity profiles were extracted from the dashed blue and green boxes, respectively. Given that the peak-to-peak height in a noise level is 1 A.U. in vacuum, the DPC signal strength obtained in the geometry of a diagonal cut is very weak (only ~2 A.U.) which is ~ 16 times smaller than the other cuts parallel to the crystal axes.

We also performed DPC imaging along the other directions to double-check the DPC results (Fig. 4). The DPC images presented in the main manuscript corresponds to the side view 1 and the planar view. The other in-plane orientation was also investigated to get the electric field component along the *y* direction (side view 2). Although the observed region is complicated by the narrow domains and deformation as shown in the figure, the electric field profile is consistent with the original DPC result taken through the side view 1. All these comprehensive efforts to rule out other influences and reconfirm the measurement results using various geometries have led to the conclusion that the flexoelectric depolarization field is present on the macro-domain walls.

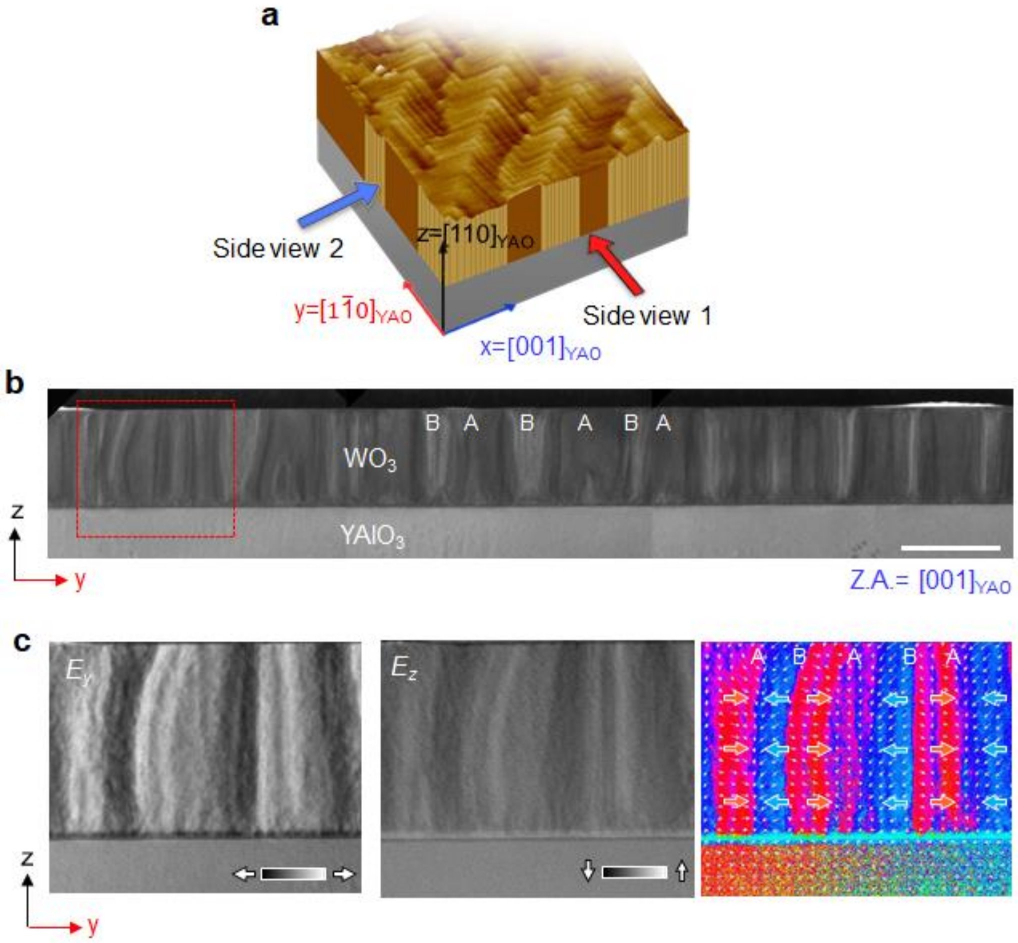

Supplementary Fig. 22 | Electric fields at the polar domain walls constructed using the DPC-STEM. **a**, Schematic of the zone-axes for the DPC-STEM imaging. **b**, The cross-sectional BF-TEM image taken along the [001] direction of $YAlO_3$ substrate. Scale bar represents 300 nm. **c**, E_y (left), E_z (center) and vector map (right) for the selected area indicated by red rectangle in (b). The horizontal components (E_y) strongly contribute to the DPC-STEM image.

Supplementary Note 10. Ferroelastic domain wall widths for different film thicknesses

To check the thickness dependency of ferroelastic domain wall width, PFM experiments were executed for four different film thicknesses (Supplementary Fig. 23). The widths were characterized by 2×FWHMs, which were obtained by Gaussian fitting the cross-sections. Distinct from the thickness (t) dependency of macro- and fine-domain widths ($w_{\mathrm{m}} \propto t^{0.6}$ and $w_{\mathrm{f}} \propto t^{0.4}$; Ref.[6]), the macro-domain 'wall' widths are almost the same as ~30 nm regardless of the different thicknesses. The spatial range of strain gradient distribution doesn't seem to be dependent on the dimension of film normal direction (z-axis), because in-plane shear strain ($\varepsilon_{x'y'}$) mainly changes across the macro-domain wall along x'-axis.

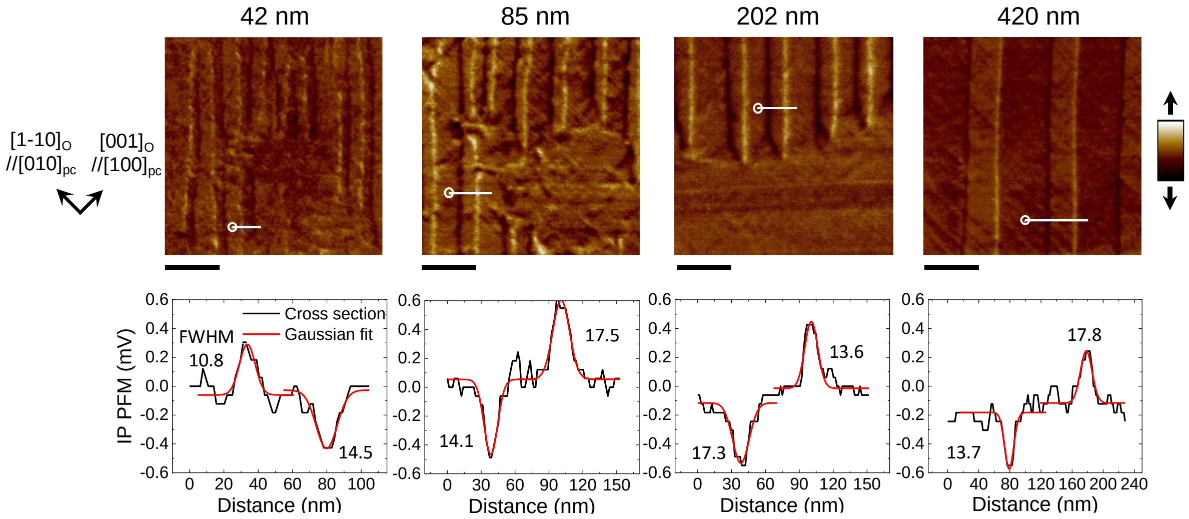

Supplementary Fig. 23 | Domain-wall width depending on the film thickness. In-plane PFM images (top) and its cross-sections (bottom) for the four different film thicknesses. White lines in the in-plane PFM images represent the cross-sections. In the cross-section graphs, values of FWHMs are indicated with the unit of nanometers. The scale bar represents the direction and relative magnitude of in-plane PFM signal acquired at the horizontal tip orientation. Scale bars indicate 200 nm.

Supplementary References

1. Woodward, P. M. & Sleight, A. W. Ferroelectric tungsten trioxide. *J. Solid State Chem.* **131**, 9-17 (1997).

2. Lee, W. T., Salje, E. K. H. & Bismayer, U. Domain-wall structure and domain-wall strain. *J. Appl. Phys.* **93**, 9890-9897 (2003).

3. Chu, K., Jang, B.-K., Sung, J. H., Shin, Y. A., Lee, E.-S., Song, K., Lee, J. H., Woo, C.-S., Kim, S. J., Choi, S.-Y., Koo, T. Y., Kim, Y.-H., Oh, S.-H., Jo, M.-H. & Yang, C.-H. Enhancement of the anisotropic photocurrent in ferroelectric oxides by strain gradients. *Nat. Nanotechnol.* **10**, 972-979 (2015).

4. Gaillac, R., Pullumbi, P. & Coudert, F.-X. ELATE: an open-source online application for analysis and visualization of elastic tensors. *J. Phys.: Condens. Matter* **28,** 275201 (2016); Gaillac, R. *ELATE: Elastic tensor analysis, stiffness matrix of* WO_3*:* http://progs.coudert.name/elate/mp?query=mp-32777.

5. Hirose, T. & Furukawa, Kazuo, Dielectric anomaly of tungsten trioxide WO_3 with giant dielectric constant. *Phys. Stat. Sol.* **203**, 608-615 (2006).

6. Yun, S., Woo, C.-S., Kim, G.-Y., Sharma, P., Lee, J. H., Chu, K., Song, J. H., Chung, S.-Y., Seidel, J., Choi, S.-Y. & Yang, C.-H. Ferroelastic twin structures in epitaxial WO_3 thin films. *App. Phys. Lett.* **107**, 252904 (2015).

7. Quang, H. L. & He, Q.-C. The number and types of all possible rotational symmetries for flexoelectric tensors. *Proc. R. Soc. A* **467**, 2369-2386 (2011).

8. Stengel, M. Surface control of flexoelectricity. *Phys. Rev. B* **90**, 201112 (2014).

9. Lohr, M., Schregle, R., Jetter, M., Wächter, C., Wunderer, T., Scholz, F. & Zweck, J. Differential phase contrast 2.0—Opening new "fields" for an established technique. *Ultramicroscopy* **117**, 7–14 (2012).

10. Shibata, N., Findlay, S. D., Sasaki, H., Matsumoto, T., Sawada, H., Kohno, Y., Otomo, S., Minato, R. & Ikuhara, Y. Imaging of built-in electric field at a p-n junction by scanning transmission electron microscopy. *Scientific Reports* **5**, 10040 (2015).
